# Structural insight into the self-activation and G-protein coupling of P2Y2 receptor

**DOI:** 10.1038/s41421-025-00797-x

**Published:** 2025-05-13

**Authors:** Baoliang Lan, Shuhao Zhang, Kai Chen, Shengjie Dai, Jiaqi Fei, Kaixuan Gao, Xiaoou Sun, Bin Lin, Xiangyu Liu

**Affiliations:** 1 State Key Laboratory of Membrane Biology, Tsinghua-Peking Center for Life Sciences, School of Pharmaceutical Sciences, Tsinghua University, Beijing, China; 2 Beijing Frontier Research Center for Biological Structure, Tsinghua University, Beijing, China; 3 Wuya College of Innovation, Shenyang Pharmaceutical University, Shenyang, Liaoning China; 4 Key Laboratory of Structure-Based Drug Design and Discovery of Ministry of Education, Shenyang Pharmaceutical University, Shenyang, Liaoning China; 5 Academy for Advanced Interdisciplinary Studies, Peking University, Beijing, China; 6 School of Basic Medicine Sciences, Tsinghua University, Beijing, China; 7 Beijing Key Laboratory of Cardiovascular Receptors Research, Peking University, Beijing, China

**Keywords:** Cryoelectron microscopy, Extracellular signalling molecules

## Abstract

Purinergic P2Y2 receptor (P2Y2R) represents a typically extracellular ATP and UTP sensor for mediating purinergic signaling. Despite its importance as a pharmacological target, the molecular mechanisms underlying ligand recognition and G-protein coupling have remained elusive due to lack of structural information. In this study, we determined the cryo-electron microscopy (cryo-EM) structures of the apo P2Y2R in complex with G_q_, ATP-bound P2Y2R in complex with G_q_ or G_o_, and UTP-bound P2Y4R in complex with G_q_. These structures reveal the similarities and distinctions of ligand recognition within the P2Y receptor family. Furthermore, a comprehensive analysis of G-protein coupling reveals that P2Y2R exhibits promiscuity in coupling with both G_q_ and G_o_ proteins. Combining molecular dynamics simulations and signaling assays, we elucidate the molecular mechanisms by which P2Y2R differentiates pathway-specific G_q_ or G_o_ coupling through distinct structural components on the intracellular side. Strikingly, we identify a helix-like segment within the N-terminus that occupies the orthosteric ligand-binding pocket of P2Y2R, accounting for its self-activation. Taken together, these findings provide a molecular framework for understanding the activation mechanism of P2Y2R, encompassing ligand recognition, G-protein coupling, and a novel N-terminus-mediated self-activation mechanism.

## Introduction

Purinergic signaling emerged in the very early stage of evolution and is omnipresent in various species, in which extracellular purines and pyrimidines are exploited to coordinate cellular function through diverse receptors, including ionotropic P2X receptors and metabotropic P1 and P2Y receptors^1^. The nucleotide-responsive P2Y receptors (P2YRs), consisting of eight members in humans, belong to δ-branch of class A rhodopsin-like G-protein coupled receptors (GPCRs)^2^. Based on distinct G-protein coupling profiles, P2YRs can be further classified into two subfamilies. The P2Y1R-like subfamily, comprising the P2Y1R, P2Y2R, P2Y4R, P2Y6R, and P2Y11R, primarily couples to G_q/11_ proteins, while the P2Y12R-like subfamily, comprising the P2Y12R, P2Y13R, and P2Y14R, primarily couples to G_i/o_ proteins^3^. Differential affinities of P2YRs for various extracellular nucleotides contribute significantly to the complexity of purinergic signaling.

Adenosine triphosphate (ATP) is one of the most widespread extracellular signaling molecules^1^. It can be released from intracellular storage pools into the extracellular compartment to mediate purinergic signaling in several physiologically relevant processes such as neurotransmission^4^, inflammation^5^ and apoptosis^6^. Among the P2YRs, the P2Y2 receptor (P2Y2R) is fully activated by extracellular ATP and UTP^7^. Initially cloned from mouse neuroblastoma cells^8^, P2Y2R is widely expressed across various human organs and tissues, with particularly higher expression levels in the skeletal muscle, heart, spleen, bone marrow, lungs and intestine^9^. Studies utilizing molecular genetics techniques have unveiled the physiological functions of the P2Y2R. Knockout studies of P2Y2R have revealed impaired endothelial nitric oxide synthase activation associated with vasodilation^10^, disrupted chloride secretion in airway epithelial cells^11^, compromised chemotaxis of neutrophils^12^, decelerated skin wound healing^13^, as well as defective cardiogenic-mesoderm induction^14^. These findings underscore the crucial roles of P2Y2R in regulating cellular functions and maintaining tissue homeostasis. The physiological functions of P2Y2R are closely associated with different G-protein-mediated downstream signaling. Similar to other P2Y1R-like receptors, P2Y2R couples to the canonical G_q/11_ protein, leading to the activation of subsequent intracellular Ca^2+^ wave propagation. Specifically, mechanistic studies have revealed that P2Y2R can also display coupling with G_12_ and G_o_ proteins through interactions with α_V_β_3/5_ integrins, thereby triggering Rho and Rac signaling pathways and influencing cell migration and cytoskeletal rearrangements that are required for chemotaxis^15–17^. Despite the significance of G-protein-relevant signaling in the physiological functions of P2Y2R, the precise molecular mechanisms underlying its distinct G-protein couplings remain poorly understood. Furthermore, several studies have shown that constitutive activity of P2Y2R is involved in regulating spontaneous endothelial sprouting in the vascular system, basal lipolysis in adipocytes, and wound healing in HaCaT keratinocyte^18–20^. To our knowledge, one explanation is that the autocrine of ATP contributes the constitutive P2Y2R signaling^19^. The existence of other endogenous stimulators in vivo that modulate the basal activity of P2Y2R remains unclear.

The pharmacological targeting of P2Y2R has gained attention due to its therapeutic potential. Dinucleotide compounds, diquafosol and denufosol, have shown promise in the management of dry eye syndrome and cystic fibrosis syndrome by stimulating chloride secretion and mucin release^21–23^. Conversely, evidence suggests that antagonism of P2Y2R can be implicated in the treatment of cancers and chronic inflammatory diseases^15^. Recent advancements in the structural determination of P2Y1R and P2Y12R, in complex with agonists or antagonists, have provided valuable insights into the orthosteric ligand binding modes for P2YRs^24–28^. However, the selectivity for nucleotides and the G-protein coupling profiles exhibit variations across different P2YRs^3,29,30^. Lack of structural information hampers our understanding of how P2Y2R recognizes its ligands and initiates distinct G-protein coupling. To address these questions, we performed structural studies, functional assays and molecular dynamics (MD) simulations to elucidate the activation mechanism of P2Y2R, with the closely related P2Y4R for comparison^31^.

## Results

### Signaling profiles and overall structures of P2Y2R and P2Y4R

**Fig. 1:**
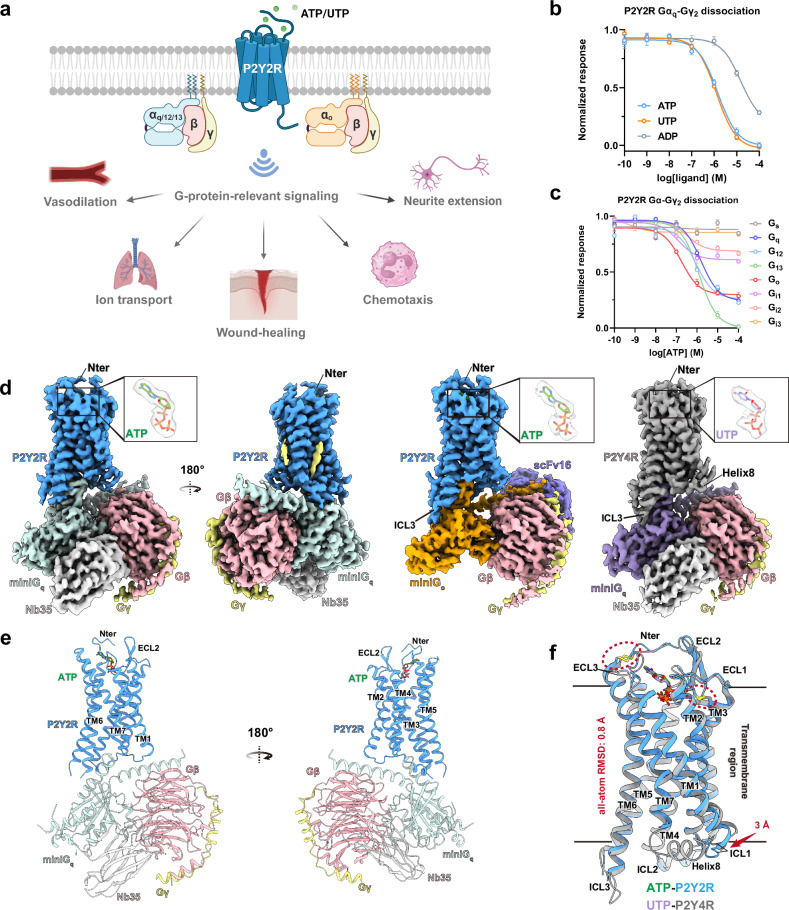
Signaling profiles and cryo-EM structures of ATP–P2Y2R–G_q_, ATP–P2Y2R–G_o_, and UTP–P2Y4R–G_q_ complexes. **a** Illustration of G-protein signaling pathways of the P2Y2R and related functions. Created with BioRender.com. **b**, **c** Dose-response curves for P2Y2R in response to distinct ligands (**b**) and its coupling to respective G-protein subtypes (**c**) measured by NanoBiT-based G-protein dissociation assay. The data represent mean ± SEM of three independent experimental replicates (*n* = 3). The fold change in luminescence value after agonist treatment, relative to the basal level, is used to reflect receptor activation and is normalized to the fraction of maximal response. A detailed statistical evaluation is listed in Supplementary Tables S1 and S2. **d** Cryo-EM density maps of the ATP–P2Y2R–miniG_q_–Nb35, ATP–P2Y2R–miniG_o_–scFv16, and UTP–P2Y4R–miniG_q_–Nb35 complexes, colored according to different subunits. ATP (green) and UTP (purple) are shown in close-up views with models fitted into their cryo-EM density maps. The map of the ATP–P2Y2R–miniG_q_–Nb35 complex is displayed from the front and back views, with two sterol-like densities highlighted in yellow. **e** Cartoon representation of the ATP–P2Y2R–miniG_q_–Nb35 complex structure from the front and back views. **f** Structural superposition of the ATP-bound P2Y2R–G_q_–Nb35 and UTP-bound P2Y4R–G_q_–Nb35 complexes. The receptors from the two structures are aligned and displayed. Other subunits are omitted for clarity. P2Y2R is colored in blue, and P2Y4R is in gray. Conformational changes of TM1 and ICL1 are indicated by red arrows, and the two disulfide bonds are highlighted within a red dashed circle.

P2Y2R is well-characterized for its crucial roles in regulating multiple physiological functions in response to endogenous ATP and UTP (Fig. 1a). To comprehensively investigate the activation of P2Y2R, we performed NanoBiT-based G-protein dissociation assays in human embryonic kidney (HEK) 293 cells to profile the signaling of P2Y2R in response to different ligands and its coupling to various G-protein subtypes. Consistent with previous pharmacological studies^7^, ATP and UTP exhibit comparable potency in activating the canonical G_q_ signaling of P2Y2R, whereas ADP displays relatively weaker potency (Fig. 1b; Supplementary Table S1). Based on the concentration-dependent curves, the G-protein coupling profiles of P2Y2R were further characterized under treatment with ATP. In the eight representative G-protein subtypes, P2Y2R exhibits promiscuous couplings, strongly engaging with G_q_, G_12_, G_13_, and G_o_, while showing no coupling or relatively weaker coupling to G_s_ and the three G_i_ subtypes: G_i1_, G_i2_, and G_i3_ (Fig. 1c; Supplementary Table S2). Notably, P2Y2R exhibits strong G_o_ coupling activity, as evidenced by the higher pEC_50_ values relative to G_q_, G_12_, and G_13_ subtypes, and a higher *E*_max_ relative to the three G_i_ subtypes. Although P2Y4R, another P2Y1R-like receptor, shares the highest sequence homology with P2Y2R^32^, it exhibits distinct nucleotide activation profiles (Supplementary Fig. S2d and Table S1). Specifically, UTP functions as a full agonist for P2Y4R, while ATP acts as a partial agonist, as demonstrated by our G_s/q_-based cAMP accumulation assay and previous pharmacological studies of P2Y4R^31^.

To elucidate the activation mechanism of P2Y2R underlying ligand recognition and G-protein coupling, we used cryogenic-electron microscopy (cryo-EM) to determine the active state structures of P2Y2R in complex with G_q_ or G_o_ heterotrimer. Of note, the engineered mini-G-proteins, namely mini-G_s/q_ and mini-G_o_, were fused to the C-terminus of the receptor to stabilize the receptor–G-protein complexes. The N-terminus sequences of G_αi1_ were introduced to the mini-G-proteins to facilitate their binding to scFv16. Purified P2Y2R-miniG_s/q_ and P2Y2R-miniG_o_ fusion proteins were incubated with Gβγ and Nb35 (or scFv16) to constitute the receptor–G-protein complex for structure determination (Supplementary Figs. S8a, f and S9a). Hereafter, G_q_ and G_o_ refer to miniG_s/q_ and miniG_o_, respectively, unless otherwise specified. After collecting cryo-EM data for these complex samples, we finally determined three active state structures of P2Y2R: the apo form in complex with G_q_ at a global resolution of 3.31 Å (Supplementary Fig. S9b–d), the ATP-bound form in complex with G_q_ at 2.83 Å (Supplementary Fig. S8b–d), and the ATP-bound form in complex with G_o_ at 2.65 Å (Supplementary Fig. S8g–i). For comparative analysis, we also applied the same strategy to determine the UTP-bound P2Y4R–G_q_ complex structure at a global resolution of 3.14 Å (Supplementary Fig. S9f–i). The cryo-EM map densities allowed for unambiguous modeling of the ligands and all subunits of the complexes (Fig. 1d; Supplementary Figs. S8e, j and S9e, j). However, the Helix 8 is disordered in all P2Y2R structures, indicating a highly flexible nature in this region (Fig. 1d, f; Supplementary Figs. S8 and S9).

**Fig. 2:**
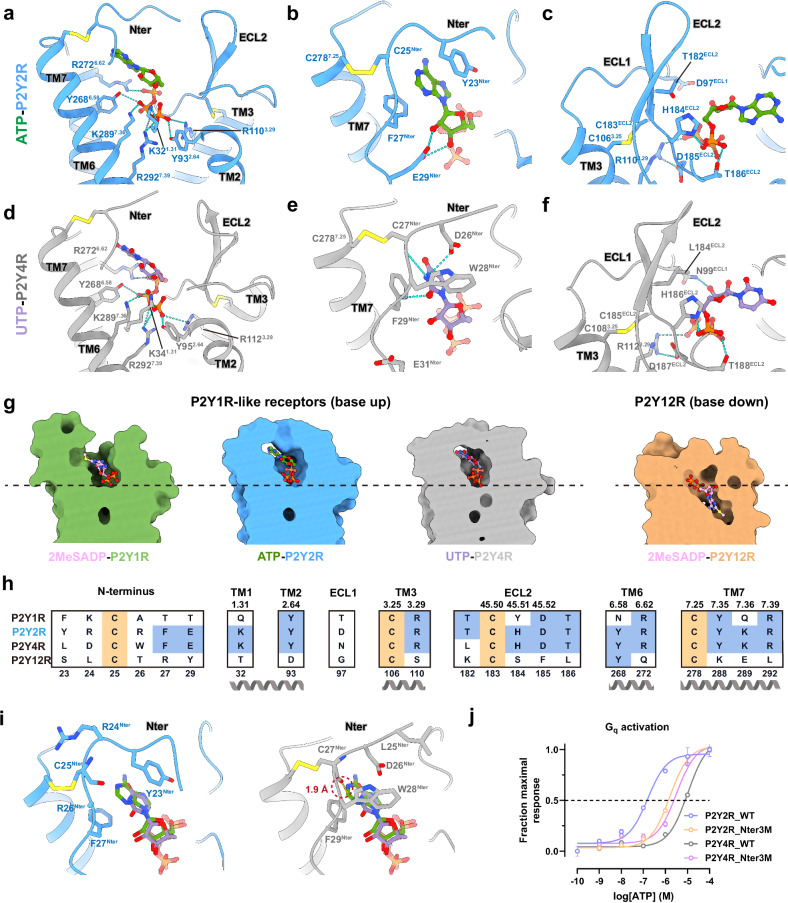
Molecular recognition of ATP and UTP by P2Y2R and P2Y4R. **a**–**c** Interactions between ATP and the transmembrane core (**a**), N-terminus (**b**), and ECL2 (**c**) of P2Y2R. Polar interactions, including hydrogen bonds and salt bridges, are depicted as light blue dashed lines. Disulfide bonds are represented as yellow sticks. **d**–**f** Interactions between UTP and the transmembrane core (**d**), N-terminus (**e**), ECL1 and ECL2 (**f**) of P2Y4R. **g** Comparison of the ligand-binding pockets of four P2YRs: P2Y1R (PDB: 7XXH), P2Y2R (this study), P2Y4R (this study), and P2Y12R (PDB: 7XXI). The ligands bound to each receptor are depicted as follows: 2MeSADP (pink) for P2Y1R and P2Y12R, ATP (green) for P2Y2R, and UTP (purple) for P2Y4R. A black dashed line drawn from the bottom of ligand-binding pocket of P2Y2R serves as a reference to compare the depth of the ligand-binding pockets of different P2YRs. **h** Sequence alignment of the ligand-binding pockets of four P2YRs: P2Y1R, P2Y2R, P2Y4R, and P2Y12R. The conserved residues are highlighted in wheat or light blue. **i** Comparison of the N-terminal sub-pockets between ATP-bound P2Y2R–G_q_ and UTP-bound P2Y4R–G_q_ structures. Receptors are aligned, and two views in the same orientation display the overlay of ATP and UTP in the sub-pocket formed by N-terminus in P2Y2R or P2Y4R. For clarity, the cartoon form of one receptor is shown while the other is hidden. **j** Mutational effects of P2Y2R_Nter3M (Y23^Nter^L-R24^Nter^D-R26^Nter^W triple mutation in P2Y2R) and P2Y4R_Nter3M (L25^Nter^Y-D26^Nter^R-W28^Nter^R triple mutation in P2Y4R) on activation potency for both receptors in response to ATP. This experiment was performed using the G_s/q_-based cAMP accumulation assay, and a detailed description is provided in Materials and Methods. The difference of luminescence intensity before and after agonist treatment is used to reflect receptor activation and is normalized to the maximal response. The dose-response curse represents the global fit of mean ± SEM from grouped data with three independent experimental replicates (*n* = 3). A detailed statistical evaluation is listed in Supplementary Table S4.

The active forms of the P2Y2R and P2Y4R, when bound to the G_q_ protein, assume similar configurations, as evidenced by an all-atom root-mean-square deviation (RMSD) of 0.8 Å for the receptor structures (Fig. 1f). The ATP and UTP are bound to the orthosteric ligand-binding pockets of P2Y2R and P2Y4R in a similar orientation (Fig. 1f). Both receptors adopt the canonical seven-transmembrane helical bundle topology (Fig. 1f). Two disulfide bonds play a critical role in constraining the conformation of the extracellular regions of both receptors (Fig. 2a–f). In P2Y2R, one bond is formed between C106^3.25^ (hereafter, the superscripts indicate Ballesteros-Weinstein numbering for GPCRs^33^) of TM3 and C183^45.50^ of extracellular loop 2 (ECL2) (Fig. 2c), a feature that is highly conserved in the orthosteric pocket across most class A GPCRs^34^. The second bond is formed between C25^Nter^ of N-terminus and C278^7.25^ of TM7 (Fig. 2b), which is highly conserved in the P2YR family as revealed by the sequence alignment and structure analysis (Fig. 2h)^24,25,28^. Two sterol-like densities are observed in the transmembrane region near the intracellular side, stabilizing the P2Y2R. One is located in the cleft formed by TM3, TM4, and TM5, while the other is attached to the surface of TM2, TM3, and TM4 (Fig. 1d, e). Similar binding positions of sterol could be observed in the active P2Y10R, a P2YR-like receptor^35^. Notably, a substantial difference between the two purinergic receptors is that the intracellular end of TM1 and intracellular loop 1 (ICL1) in P2Y2R show displacement toward the center of transmembrane helix bundles by ~3 Å compared to P2Y4R (measured from the Cα atom of C60^1.59^ in P2Y2R to the Cα atom of F62^1.59^ in P2Y4R) (Fig. 1f). This observed displacement could be attributed to the distinct interactions between ICL1 and the G_q_ protein in P2Y2R compared to P2Y4R, which will be discussed in more detail in a subsequent section.

### Activated states of P2Y2R

Given the unavailability of the inactive state structures of P2Y2R and P2Y4R, we utilized the inactive state structure of MRS2500-bound P2Y1R (PDB: 4XNW)^24^ as a reference to investigate the activation mechanisms of the purinergic receptors P2Y2R and P2Y4R. P2Y1R shares close phylogenetic homology with P2Y2R and P2Y4R and is similarly capable of coupling with G_q/11_ proteins^28^. Relative to the inactive state of P2Y1R, P2Y2R adopts an outward movement of TM6 by ~10.7 Å for coupling with G_q_ and by 9.4 Å for coupling with G_o_ (measured from the Cα atom of residue R238^6.28^ in P2Y2R to the Cα atom of P253^6.28^ in P2Y1R), and P2Y4R adopts an outward movement of TM6 by ~10.4 Å for coupling with G_q_ (measured from the Cα atom of residue S238^6.28^ in P2Y4R to the Cα atom of P253^6.28^ in P2Y1R) (Supplementary Fig. S1b). The micro-switches, including C^6.47^-W(F)^6.48^-x-P^6.50^, P^5.50^-I(V)^3.40^-F^6.44^, D(H)^3.49^-R^3.50^-Y(C)^3.51^, and N(D)^7.49^-P^7.50^xxY^7.53^, assume similar conformational rearrangements during the activation of P2Y2R and P2Y4R (Supplementary Fig. S1d). To reveal the activation characteristics of P2Y2R when coupled with the G_q_ protein, we focus on identifying key structural elements that facilitate this process. We found that clustered residues within TM6 and TM7 are crucial for P2Y2R activation. Specifically, N285^7.32^ and Y288^7.35^ undergo a downward shift due to the tight packing of ATP, the N-terminus, and TM7 (Supplementary Fig. S1a). These conformational changes subsequently lead to the downward movement of F261^6.51^ (Supplementary Fig. S1a). The perturbation on the extracellular side of TM6 and TM7 induces a twist in F254^6.44^ of the conserved P^5.50^-I^3.40^-F^6.44^ motif, facilitated by the structural arrangement of the toggle switch residue F258^6.48^ from the C^6.47^-W(F)^6.48^-x-P^6.50^ (Supplementary Fig. S1a, d). Collectively, these coordinated structural adjustments in TM6 and TM7 ultimately drive the outward displacement of TM6 on the intracellular side, a hallmark of GPCR activation^36^. Similar activation mechanisms have also been observed in the 2MeSADP–P2Y1R–G_11_ and lysophosphatidylserine (LysoPS)–P2Y10–G_13_ complexes^28,35^. The importance of N285^7.32^, Y288^7.35^, and F261^6.51^ in the activation of P2Y2R is evident from the significant reduction in ATP-induced G_q_ activation potency in the corresponding alanine mutants (Supplementary Fig. S1c and Table S3). Notably, P2Y2R features the H^3.49^-R^3.50^-C^3.51^ motif rather than the conserved D^3.49^-R^3.50^-Y^3.51^ motif found in most class A GPCRs (Supplementary Fig. S1d)^37^. The inward displacement of TM5 and TM7 contributes to the direct hydrogen bond interaction between Y306^7.53^ and Y218^5.58^, with Y218^5.58^ further stabilizing the orientation of R131^3.50^ within the H^3.49^-R^3.50^-C^3.51^ motif via a hydrogen bond (Supplementary Fig. S1d). These structural cooperations collectively facilitate the formation of the binding cavity necessary for G-protein coupling of P2Y2R.

### Recognition of ATP by P2Y2R

The ATP binding mode in the P2Y2R–G_q_ complex closely resembles that in the P2Y2R–G_o_ structure (Supplementary Fig. S4a). We thus selected the P2Y2R–G_q_ complex to study the recognition of ATP by P2Y2R. The P2Y2R adopts a relatively shallow ATP-binding pocket formed by residues from transmembrane helices TM1, TM2, TM3, TM6, TM7, as well as N-terminus and ECL2 (Fig. 2a–c). The N-terminus of P2Y2R, spanning residues from L21 to E29, folds into a loop configuration (Fig. 2b). It traverses the transmembrane helix bundle under the constraint of a disulfide bond formed by C25^Nter^ and C278^7.25^ and is in proximity to the tip of ECL2, thereby defining the upper boundary of ligand-binding pocket (Fig. 2a, b). Breaking the formation of this disulfide bond by alanine mutation of C25^Nter^ nearly abolished the G_q_ activation of P2Y2R (Supplementary Fig. S1c and Table S3). The ECL2 of P2Y2R adopts a 20-residue β-hairpin structure. The upper segment of ECL2, comprising residues from V172 to C183, protrudes into the extracellular side (Fig. 2c). In contrast, the lower segment of ECL2, comprising residues from C183 to L191, inserts deeply into the ligand-binding cavity, interacting with the 5′-triphosphate moiety of ATP (Fig. 2c). The conformation of ECL2 is largely constrained by the disulfide bond between C106^3.25^ and C183^ECL2^, as well as a salt bridge between R110^3.29^ and D185^ECL2^ (Fig. 2c).

Unlike P2Y1R, ATP binds to the ligand-binding pocket of P2Y2R in a distinctly vertical orientation (Supplementary Fig. S2a). The negatively charged 5′-triphosphate moiety is nestled at the base of the orthosteric pocket, forming extensive polar contacts with the transmembrane helix core and ECL2 (Fig. 2a, c; Supplementary Fig. S2e). Specifically, the γ-phosphate group is engaged in a network of polar interactions with positively charged residues, including K32^1.31^, R110^3.29^ and R292^7.39^, forming close contacts from multiple directions (Fig. 2a). Residue Y93^2.64^ further stabilizes this phosphate group via a hydrogen bond (Fig. 2a). In contrast, ADP’s lack of the third phosphate group results in weaker interactions with the corresponding residues of P2Y2R, which likely underlies its reduced activation potency compared with ATP (Fig. 1b; Supplementary Table S1). The β-phosphate group is recognized by Y268^6.58^ and K289^7.36^ through hydrogen bonding and salt bridge interactions (Fig. 2a). The α-phosphate group mainly interacts with the side chains of H184^ECL2^ and T186^ECL2^ in the embedded ECL2 (Fig. 2c). It is noteworthy that the pocket residues Y93^2.64^, R110^3.29^, R272^6.62^, and R292^7.39^ from the transmembrane helix of P2Y2R are also highly conserved in the ligand-binding pocket of P2Y1R and P2Y4R as revealed by the sequence alignment and structure analysis (Fig. 2h; Supplementary Fig. S2a). This conservation highlights a shared ligand recognition mechanism in the P2Y1R-like subfamily. Consistent with the structural observations, alanine substitution of these pocket residues markedly impairs ATP-induced P2Y2R activation of G_q_ or G_o_, as indicated by the G-protein dissociation assay in HEK293T cells expressing P2Y2R (Supplementary Fig. S5a–f, j and Table S5). These results suggest that the interactions between the transmembrane helix and ECL2 of P2Y2R and the 5′-triphosphate moiety of ATP are crucial for receptor activation. The critical role of the triphosphate group is further underscored by previous functional studies employing substitution analogs at P2Y2R. For example, R_p_-α-S-UTP (α-thio substitution of UTP)^38^, ATPγS (γ-thio substitution of ATP)^7^, and UppCF_2_p (β,γ-difluoromethylene substitution of UTP)^39^ exhibit significantly lower potencies compared to their corresponding endogenous ligands.

In addition to the recognition of the 5′-triphosphate moiety by P2Y2R, the base and ribose moieties are also specifically recognized. Notably, a part of loop segment in the N-terminus shapes as a ‘cap’ to cover the ligand-binding pocket, comprising residues Y23^Nter^, C25^Nter^, F27^Nter^ and E29^Nter^, which collectively stabilize the base and ribose moieties of ATP in P2Y2R (Fig. 2b; Supplementary Fig. S2e). Specifically, residues Y23^Nter^ and F27^Nter^ form π–π interactions with the protruded adenine moiety, which are sandwiched by these two aromatic residues (Fig. 2b). In addition, the side chain of E29^Nter^ forms a hydrogen bond interaction with a hydroxyl group of ribose. Similarly, a series of substitutions at the ribose ring of UTP, including 2′-deoxy-UTP, 2′-azido-2′-deoxy-UTP, 2′-deoxy-2′-fluoro-UTP, and 2′-deoxy-2′-methoxy-UTP^38^, exhibit lower potency in P2Y2R activation compared to UTP. The importance of the interactions between the N-terminus and ATP is further supported by G-protein dissociation assay. Alanine mutations at positions Y23^Nter^, F27^Nter^, and E29^Nter^ lead to significant reductions in G_q_ or G_o_ activation potency for P2Y2R (Supplementary Fig. S5a, j and Table S5).

### Comparison of nucleotide recognition modes across P2YRs

Compared with the ATP-bound P2Y2R–G_q_ complex structure, P2Y4R exhibits a generally similar extracellular arrangement in its UTP-bound G_q_ complex structure, encompassing the N-terminus, ECL2, and transmembrane helices (Fig. 2a–f). The residues lining the ligand-binding pocket display a high degree of conservation across several P2YRs, as evidenced by sequence alignment (Fig. 2h). As a result, similar to the interactions in P2Y2R, an extensive polar network is also formed between the 5′ triphosphate moiety of UTP and P2Y4R, involving residues K34^1.31^, Y95^2.64^, R112^3.29^, Y268^6.58^, R272^6.62^, K289^7.36^, and R292^7.39^ from the transmembrane helix core, as well as H186 and T188 from ECL2 (Fig. 2d, f; Supplementary Fig. S2e). The only difference is that it is N99^ECL3^ in P2Y4R instead of E29^Nter^ in P2Y2R which stabilizes the ribose by a hydrogen bond (Fig. 2f; Supplementary Fig. S2e). Disruption of the above polar interactions by alanine replacement of these key residues significantly weakens UTP-induced P2Y4R G_q_ activation potency, as revealed by the G_s/q_-based cAMP accumulation assay (Supplementary Fig. S5g–i and Table S5).

Structural analysis of the ligand-binding pocket of P2YRs reveals both shared and unique nucleotide recognition modes. The P2Y1R, P2Y2R, and P2Y4R receptors share a superficial binding pocket, wherein the ligands adopt a ‘base-up’ orientation (Fig. 2g; Supplementary Fig. S2c). In contrast, the P2Y12R adopts a deeper and more sealed ligand-binding pocket, wherein the base moiety adopts a downward ‘base-down’ orientation (Fig. 2g; Supplementary Fig. S2c). ATP establishes polar contacts with TM1 and TM2 but is distant from TM4 and TM5 in P2Y2R. In contrast, 2-methylthio-ADP (2MeSADP) binding to P2Y12R exhibits an opposing orientation, with closer proximity to TM4 and TM5 and reduced interactions with TM1 and TM2 (Supplementary Fig. S2c). These findings suggest that P2Y1R-like receptors and P2Y12R-like receptors have distinct topological features in their orthosteric ligand-binding pocket. In the P2Y1R-like subfamily, residues Y^2.64^, R^3.29^, R^6.62^, and R^7.39^ in P2Y1R, P2Y2R, and P2Y4R are involved in conserved interactions with the phosphate moiety of nucleotides (Supplementary Fig. S2a). Compared with 2MeSADP-bound P2Y1R, ATP and UTP in P2Y2R and P2Y4R undergo an ~180° flip, contributing to the closer packing of their 5′-triphosphate moieties with TM1 and the N-terminus (Supplementary Fig. S2b). For instance, in P2Y2R, the adenine moiety of ATP interacts with Y23^Nter^ and F27^Nter^, while the ribose and 5′-triphosphate group form polar contacts with E29^Nter^ and K32^1.31^, respectively (Supplementary Fig. S2b). These extensive interactions between the N-terminus and ATP represent a ligand binding mode distinct from P2Y1R, wherein no interactions between the N-terminus and 2MeSADP are observed. In P2Y1R, the adenine of ATP interacts with Y303^7.32^, and the 5′-diphosphate moiety is in proximity to ECL2 (Supplementary Fig. S2b). These structural analyses reveal both common and distinct ligand recognition modes for P2YRs.

It is well established that the P2YRs display promiscuous nucleotide binding, characterized by distinct activation profiles and potencies (Fig. 1b; Supplementary Fig. S2d and Table S1)^29,30^. The pharmacological characterization of human P2Y2R and P2Y4R were previously performed after stable transfection into 1321N1 astrocytoma cells. ATP functions as a full agonist for P2Y2R but as a partial agonist for P2Y4R^7,31^. Consistently, our functional assays demonstrate that both ATP and UTP are capable of fully activating P2Y2R with comparable potency and efficacy, whereas ATP exhibits lower potency and efficacy in the activation of P2Y4R compared to the full agonist UTP (Fig. 1b; Supplementary Fig. S2d and Table S1). These findings prompted us to investigate the mechanism underlying the preferential recognition of ATP by P2Y2R over P2Y4R. Structure comparison and sequence alignment reveal that the most substantial difference in ligand-binding pocket between P2Y2R and P2Y4R lies in the N-terminus, which recognizes the base moiety of ATP or UTP (Fig. 2h, i). Unlike P2Y2R, wherein the adenine of ATP is sandwiched between Y23^Nter^ and F27^Nter^, the uracil of UTP in P2Y4R fits into a narrower sub-pocket formed by D26^Nter^, W28^Nter^, and F29^Nter^ (Fig. 2i). Structure overlay between ATP-bound P2Y2R and UTP-bound P2Y4R structures suggests that the sub-pocket of N-terminus in P2Y2R is capable of accommodating both ATP and UTP, whereas the sub-pocket of N-terminus in P2Y4R disfavors ATP recognition due to the steric clash between the adenine moiety and the backbone carbonyl oxygen of C27^Nter^ (Fig. 2i). The distinct interaction patterns in the N-termini of P2Y2R and P2Y4R suggest that this region contributes significantly to the receptors’ ligand recognition specificity and affinity. To corroborate this observation, we performed mutagenesis studies on the N-termini of P2Y2R and P2Y4R, swapping three key residues between these two receptors (Fig. 2h–j; Supplementary Table S4). The P2Y2R_Nter3M mutant is generated by introducing Y23L, R24D and R26W mutations, while the P2Y4R_Nter3M mutant is generated by introducing L25Y, D26R and W28R mutations. Consistent with our hypothesis, the P2Y4R_Nter3M mutant exhibits enhanced G_q_ activation potency compared to the wild-type (WT) P2Y4R in response to ATP, while the P2Y2R_Nter3M mutant exhibits reduced G_q_ activation potency compared to P2Y2R in response to ATP (Fig. 2j), highlighting the important role of the N-terminus in determining the ATP recognition specificity of P2Y2R and P2Y4R.

### G_q_ coupling mechanism of P2Y2R and other P2Y1R-like receptors

**Fig. 3:**
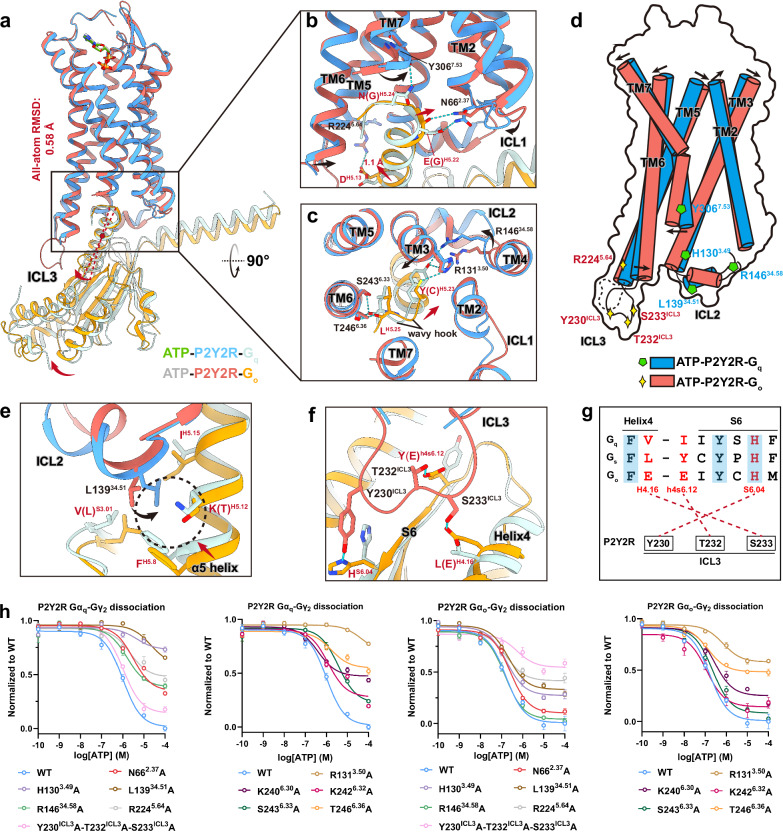
Molecular determinants of the P2Y2R G_q_ and G_o_ couplings. **a** Structural superimposition of ATP–P2Y2R–G_q_ and ATP–P2Y2R–G_o_ complexes. The red arrows indicate the conformational changes of miniG_q_ and miniG_o_ proteins when aligning the receptors of the two structures. **b**, **c** Close-up representations of the interfaces between the P2Y2R and the α5 helices of G_q_ and G_o_ proteins in side view (**b**) and top view (**c**). The black arrows indicate the conformational changes of P2Y2R when coupling to G_q_ or G_o_ protein. The red curved arrows indicate the rotational shift of the α5 helix from G_q_ to G_o_. The side chains of residues that exhibit different interactions in P2Y2R–G_q_ or –G_o_ interfaces are depicted as sticks. Polar interactions are represented as light blue dashed lines. Hereafter, for G-proteins, the residues labeled outside the parentheses correspond to G_q_, and the ones inside correspond to G_o_. **d** Schematic representation of the conformational changes of P2Y2R and key interface residues that display distinct interactions in G_q_ and G_o_ couplings. The residues that specifically interact with G_q_ are depicted as green pentagons, whereas the residues that specifically interact with G_o_ are depicted as yellow four-pointed star. **e** Hydrophobic interactions of residue L139^34.51^ in ICL2 with G_q_ and G_o_ proteins. The rotational shift of residue L139^34.51^ from the G_o_-coupled state to the G_q_-coupled state is indicated by a black curved arrow. **f** Polar interactions between ICL3 of the receptor and G_o_ proteins. The hydrogen bond interactions between ICL3 and G_o_ are shown as blue dashed lines, with the corresponding residues depicted as sticks. **g** Schematic representation of specific interactions between ICL3 and G_o_ protein. In the sequence alignment of G_o_, G_q_, and G_s_ proteins, interaction residues in G_o_ and their equivalents in G_q_ and G_s_ are highlighted in red. Conserved residues across different G-protein subtypes are indicated by a light blue background. **h** Effects of alanine mutations of residues in the P2Y2R–G-protein interfaces on Gα_q_–Gγ_2_ or Gα_o_–Gγ_2_ dissociation induced by ATP, compared to WT receptor. The dose-response curves represent the global fit of mean ± SEM from grouped data with three independent experimental replicates (*n* = 3). A detailed statistical evaluation is listed in Supplementary Table S6.

The P2Y1R-like subfamily, encompassing P2Y1R, P2Y2R, P2Y4R, P2Y6R, and P2Y11R, is characterized by the capability for G_q_ coupling^3^. Generally, P2Y2R adopts a G_q_ coupling mode similar to P2Y1R and P2Y4R. The TM2, TM3, TM5, TM6, TM7, as well as ICL2 in the receptors, create the binding cavity for the G_q_ protein (Fig. 3a–c; Supplementary Fig. S3 and Table S15). In the P2Y2R–G_q_ complex structure, P2Y2R adopts a series of interactions that are conserved in P2Y1R-like receptors to accommodate the α5 helix of G_q_ (Supplementary Fig. S3d). For instance, the side chains of H130^3.49^ and R131^3.50^ in H^3.49^-R^3.50^-C^3.51^ motif form hydrogen bonds with the side chain and backbone carbonyl of Y^H5.23^ in G_q_, respectively, while the backbone carbonyls of G134^3.53^ and Y306^7.53^ in the receptor establish hydrogen bond interactions with the side chains of N^H5.19^ and N^H5.24^ in G_q_, respectively. In addition, residues V135^3.54^, L225^5.65^, M221^5.61^, and I247^6.37^ of P2Y2R are also engaged in series of van der Waals interactions with the hydrophobic core composed of L^H5.25^, L^H5.20^, and L^H5.16^ in the α5 helix (Supplementary Fig. S3d). Notably, L139^34.51^ in P2Y2R protrudes into a hydrophobic groove formed by I^H5.15^, K^H5.12^ and F^H5.8^ of the α5 helix and V^S3.01^ of the β2–β3 loop in G_q_ (Supplementary Fig. S3c). The interactions between L139^34.51^ and the α5 helix of G_q_ are conserved across reported P2Y1R-like receptors, suggesting a common G_q_ coupling mechanism within this subfamily (Supplementary Fig. S3b, c). While in P2Y12R, the counterpart in position 34.51 is the bulky residue F^34.51^ instead of L^34.51^, which pushes the α5 helix of G_i2_ to move toward TM5 compared with G_q_ in P2Y1R, P2Y2R and P2Y4R (Supplementary Fig. S3a). The importance of the above key residues in G-protein coupling is demonstrated by mutagenesis studies of the receptor, wherein alanine replacements of H130^3.49^, R131^3.50^, or L139^34.51^ significantly impair the dissociation of Gα_q_ and Gγ_2_, as revealed by lower efficacy or potency relative to the WT P2Y2R in response to ATP (Fig. 3h; Supplementary Table S6).

The Gα subunit mainly engages the receptor through the C-terminus of α5 helix^40^. The extreme C-terminus of Gα, often referred to as the ‘wavy hook’, forms an extra α-helical-turn extending from the α5 helix upon interaction with a receptor^41–43^. One notable characteristic for P2Y2R–G_q_ complex is the extensive interactions between the TM6 of the receptor and the α5 helix and the ‘wavy hook’ (N^H5.24^-L^H5.25^-V^H5.26^) of G_q_ (Supplementary Fig. S3e and Table S15). In detail, the side chains of K242^6.32^, S243^6.33^ and T246^6.36^ of the receptor establish hydrogen bond interactions with the backbone carbonyl of L^H5.25^ in G_q_. The K240^6.30^ from TM6 establishes polar contacts with D^H5.13^ in G_q_. Additionally, the side chains of A239^6.29^ and I247^6.37^ form hydrophobic interactions with V^H5.26^ and L^H5.25^, respectively. These extensive interactions between TM6 and G_q_ may contribute to a smaller outward displacement of TM6 in P2Y2R compared to that observed in G_q_-coupled P2Y1R and G_i2_-coupled P2Y12R (Supplementary Fig. S3a, e). Another unique interaction in the P2Y2R–G_q_ complex involves the hydrogen bond between E^H5.22^ and N66^2.37^ in ICL1. In the P2Y1R–G_11_ complex, S84^2.37^, rather than N66^2.37^, establishes a similar hydrogen bond with E^H5.22^. However, in the P2Y4R–G_q_ complex, this hydrogen bond is lost because the corresponding residue is a negatively charged residue D68^2.37^ in the P2Y4R (Supplementary Fig. S3e), which explains the outward displacement of ICL1 in P2Y4R compared to P2Y2R (Fig. 1f). Compared to P2Y1R and P2Y4R, R146^34.58^ from ICL2 in P2Y2R forms an additional hydrogen bond with Y^H5.23^ of G_q_ (Supplementary Fig. S3e). These differences account for the distinct intracellular conformations of P2Y2R compared with other P2Y1R-like receptors. Alanine mutations of N66^2.37^, R146^34.58^, K240^6.30^, K242^6.32^, S243^6.33^, and T246^6.36^ significantly decrease the potency or efficacy of P2Y2R G_q_ activation in response to ATP (Fig. 3h; Supplementary Table S6), further supporting their crucial role in P2Y2R G_q_ activation.

### Distinct G_q_ and G_o_ coupling mechanisms in P2Y2R

Besides G_q_, P2Y2R exhibits promiscuity in coupling with other G-protein subtypes. This raises important questions about the distinct mechanisms underlying its interactions with different G-protein partners. Notably, P2Y2R demonstrates higher potency in G_o_ activation compared to G_q_ in response to ATP, as revealed by the G-protein dissociation assay (Fig. 1c; Supplementary Table S2). Mechanistic studies have shown that P2Y2R’s G_o_-mediated signaling cascade triggers cytoskeletal rearrangements, which are essential for chemotaxis and neurite extension^16,44^. To elucidate the molecular determinants for G_o_ and G_q_ couplings in P2Y2R, we compared the structures of ATP–P2Y2R–G_q_ and ATP–P2Y2R–G_o_ complexes (Fig. 3a–c). Although the receptor adopts a similar global conformation revealed by an all-atom RMSD of 0.58 Å in two P2Y2R–G-protein complexes (Fig. 3a), subtle transmembrane helix movements can be detected from MD simulations (Supplementary Fig. S4a, b). Compared with G_q_-coupled P2Y2R, the distance between TM6 and TM1 (Y268^6.58^–K32^1.31^) is narrowed in G_o_-coupled P2Y2R, which drives ATP to move slightly toward TM4 and TM5. The ATP binding pushes against ECL2, causing it to move outward along with the extracellular side of TM4 and TM5, which further drives the outward shift of TM3 via the disulfide bond formed by C106^3.25^ and C183^ECL2^ (Supplementary Fig. S4a). The changes in the ligand-binding pocket are consistent with the longer distances between TM7 and TM3 (C278^7.25^–C106^3.25^), TM7 and TM4 (C278^7.25^–F171^4.63^), and TM7 and TM5 (C278^7.25^–F192^5.32^), and shorter distances between TM6 and TM1 (Y268^6.58^–K32^1.31^) in G_o_-coupled P2Y2R relative to its G_q_-coupled form, as revealed by MD simulations (Supplementary Fig. S4b). The conformational change of TM3 in the ligand-binding pocket is likely associated with an inward movement in its intracellular region to the α5 helix of G_o_ (Fig. 3c). The effects of alanine mutations of pocket residues on G_q_ or G_o_ coupling of P2Y2R are reflected by the G-protein dissociation assay (Supplementary Fig. S5a–f, j and Table S5). However, majority of these residues are of equal importance to either G_q_ or G_o_ coupling, suggesting that the architecture of the ligand-binding pocket in P2Y2R unlikely accounts for the G-protein coupling selectivity.

The intracellular half of the receptor displays notable conformational changes between the P2Y2R–G_q_ and P2Y2R–G_o_ complexes, particularly in TM6, TM7, ICL2, and ICL3, which are cooperated by the distinct orientations of G_o_ and G_q_ relative to the receptors (Fig. 3a–c). Compared with G_q_, the α5 helix of G_o_ undergoes a 1.1 Å rotational shift (determined by the distance shift of D^H5.13^) toward TM5 when aligning the receptors (Fig. 3b). Meanwhile, the distal C-terminus of G_o_ shifts closer to TM3 and ICL2 (Fig. 3b, c). Given that the α5 helix of the G-protein provides the main binding interfaces for GPCRs, we compared the specific interactions between the α5 helices of G_o_ and G_q_ and the receptors through structural analysis and MD simulations to identify the molecular determinants for G_o_ and G_q_ coupling of P2Y2R (Fig. 3a–c; Supplementary Fig. S4c and Table S15). Overall, most of the interfaces between the receptor and the α5 helices of the G-proteins are conserved in both complex structures, explaining the promiscuity of P2Y2R in coupling with G_q_ and G_o_ (Supplementary Table S15).

Inspection of the interfaces between the receptors and G-proteins reveal specific residues that are associated with G_o_ vs G_q_ coupling (Fig. 3a–d). The C^H5.23^ of G_o_ requires a smaller space to accommodate its side chain relative to the bulky residue Y^H5.23^ in G_q_, allowing it to move toward the TM3 and ICL2. Consequently, this shift enables C^H5.23^ of G_o_ to form polar contacts with H130^3.49^ from TM3, cooperated by the inward displacement of ICL2 and TM3 (Fig. 3c). Due to a 1.1 Å rotational shift of D^H5.13^ toward TM5 in G_o_ relative to G_q_, D^H5.13^ forms a salt bridge with R224^5.64^ from TM5, whereas the equivalent interaction is not observed in the P2Y2R–G_q_ complex structure (Fig. 3b). It is corroborated by MD simulations that the distance between D^H5.13^ and R224^5.64^ is suitable to form a polar interaction in the G_o_-coupled P2Y2R, while it is too far to form a polar interaction in the G_q_-coupled P2Y2R (Supplementary Fig. S4c). However, compared with WT P2Y2R, mutant R224^5.64^A of P2Y2R displays comparable efficacy reduction in the ATP-induced G_o_ or G_q_ activation (Fig. 3h; Supplementary Table S6). Therefore, although R224^5.64^ is prone to forming interactions with G_o_ rather than G_q_, it is not responsible for the specificity of G_o_ coupling. Another special feature in G_o_-coupled P2Y2R is ICL3. In the cryo-EM map of the P2Y2R–G_o_ complex structure, the density of ICL3 is well-defined, whereas the density is disordered in the P2Y2R–G_q_ complex structure due to its flexibility (Figs. 1d, e and 3a). A similar phenomenon has also been reported for the SSTR2, a peptide-bound GPCR that also exhibits both G_q_ and G_o_ coupling^45^. In the P2Y2R–G_o_ complex, the ICL3 engages tightly with the Ras domain of G_o_ and provides a larger binding surface for it. Specifically, residues Y230^ICL3^, T232^ICL3^, and S233^ICL3^ engage in a series of polar interactions with H^S6.04^ of β6, E^h4s6.12^ between β6 and Helix 4, and E^H4.16^ of Helix 4 in G_o_, respectively (Fig. 3f). According to the sequence alignment of equivalent positions in G-proteins (Fig. 3g), the H^S6.04^ residues are conserved in both G_q_ and G_o_. However, E^h4s6.12^ and E^H4.16^ in G_o_ are replaced by hydrophobic residues I^h4s6.20^ and V^H4.16^ in G_q_ (Y^h4s6.20^ and L^H4.16^ in G_s_), respectively, which disfavor the formation of polar contacts. Notably, a triple-residue mutant, Y230/T232/S233^ICL3^A, shows 54% reduction in G_o_ activation efficacy and 14% reduction in ATP-induced G_q_ activation efficacy (Fig. 3h; Supplementary Table S6). Similarly, significant reduction in efficacy or potency can be observed in the GALR1 and SSTR2 when key residues in its ICL3 that engage contacts with G_o_ are mutated^45,46^. Taken together, these results suggest that the ICL3 in P2Y2R plays a more pronounced role in G_o_ coupling relative to G_q_ and thereby contributes to its G-protein coupling specificity.

Conversely, the P2Y2R exhibits specific interactions with G_q_ relative to G_o_. The G_q_-specific residue N^H5.24^ forms a hydrogen bond with the backbone carbonyl of Y306^7.53^. This interaction contributes to the displacement of the intracellular side of TM7 towards the α5 helix of the G_q_ (Fig. 3b). However, this polar contact cannot be formed in the G_o_-coupled P2Y2R due to the replacement of the counterpart residue by G^H5.24^ (Fig. 3b; Supplementary Table S14). Instead of the polar contact between C^H5.23^ of G_o_ and H130^3.49^, the bulky residue Y^H5.23^ in G_q_ appears to make a stronger contact with H130^3.49^, as indicated by the distance distribution in MD simulations (Fig. 3c; Supplementary Fig. S4c). The importance of H130^3.49^ in determining the G_q_ coupling specificity is validated by mutational studies (Fig. 3h; Supplementary Table S6). In detail, the H130^3.48^A variant shows pronounced effects on G_q_ activation, with a 71% reduction in efficacy and a 4.4-fold reduction in potency, whereas its effect on G_o_ activation is limited. Considering the notable shift of ICL2 in the G_q_-coupled P2Y2R relative to its G_o_-coupled form (Fig. 3c), we further analyzed the contribution of ICL2 to G_q_ coupling. The ICL2 in G_q_-coupled P2Y2R is stabilized by a hydrogen bond between Y^H5.23^ and R146^34.58^ in ICL2 toward the TM4 side, whereas the interaction between C^H5.23^ in G_o_ and R146^34.58^ is lost (Fig. 3c). On the other side of ICL2 toward TM3, L139^34.51^ sits in a hydrophobic pocket formed by F^H5.08^, K^H5.12^, and I^H5.15^ from the α5 helix in G_q_-coupled P2Y2R (Fig. 3e). However, in the G_o_-coupled form, these interactions are weaker due to the displacement of L139^34.51^ (Fig. 3e). It is supported by the shorter distances for pairs L139^34.51^–F^H5.8^, L139^34.51^–K^H5.12^, and L139^34.51^–I^H5.15^ in the G_q_-coupled form compared to the G_o_-coupled form through MD simulations (Supplementary Fig. S4c). Indeed, similar strong hydrophobic interactions are also formed in some reported G_q_- or G_s_-coupled GPCRs, such as β_2_AR and M1R and GALR2^46–48^, whereas these interactions are relatively weaker in some reported G_i/o_-coupled GPCRs, such as μOR, M2R, and GALR1^46,48,49^. It is not surprising that the L139^34.51^A variant demonstrates significant reduction in efficacy and potency for G_q_ activation compared with the WT P2Y2R but shows less pronounced effect on the efficacy and potency for G_o_ activation (Fig. 3h; Supplementary Table S6). The R146^34.58^ variant exhibits a 37% reduction in efficacy for G_q_ activation, with no discernible impact on G_o_ activation (Fig. 3h; Supplementary Table S6). Therefore, the sequence variations in G_q_ and its distinct interactions with the ICL2 of P2Y2R account for the G_q_ coupling specificity compared to G_o_ in P2Y2R.

Taken together, our findings reveal distinct features on both the extracellular and intracellular sides of the P2Y2R–G_o_ and P2Y2R–G_q_ complexes. The structural components on the intracellular side responsible for G_q_ or G_o_ coupling are further identified (Fig. 3d), providing the different G-protein coupling mechanism of P2Y2R.

### N-terminus leads to self-activation of P2Y2R

**Fig. 4:**
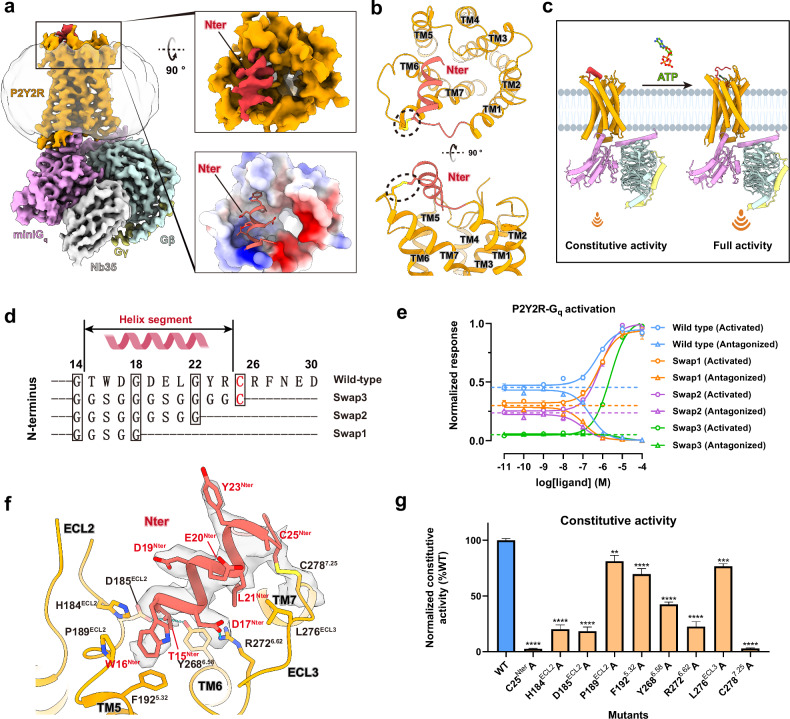
N-terminus-induced self-activation of P2Y2R. **a** Cryo-EM density map of P2Y2R–G_q_ complex in apo state with a ‘built-in’ N-terminus (Red). The black box highlights the N-terminus region, and a close-up view is shown on the right. The top right graph depicts the cryo-EM density map of the N-terminus (red) and other regions of the receptor (orange) from a top view. The bottom right graph depicts the electrostatic potential of the N-terminus binding surface, which is colored from red (negative charge) to blue (positive charge). **b** Cartoon representation of the top and side views of P2Y2R in its apo state, showing the overall interaction mode of the N-terminus (red) and other regions of P2Y2R (orange). The disulfide bond linking the N-terminus and TM7 is depicted as yellow sticks. **c** Schematic representation of N-terminus-induced self-activation and ATP-induced full activation of receptor. **d** Protein sequence of the helix-like segment in the P2Y2R N-terminus, featuring three parts spaced by glycine. The corresponding replacements of three grouped residues by a ‘GS’ linker progressively created three mutants: swap1, swap2, and swap3. **e** Effects of the three swapped mutants of N-terminus (**d**) on the constitutive activity of P2Y2R, according to a G_s/q_-based cAMP accumulation assay. The constitutive activity of the P2Y2R is assessed using a potent agonist (ATP) and antagonist (AR-C 118925XX), with luminescence values normalized to 100% for the highest ATP concentration and 0% for the highest AR-C 118925XX concentration. Each data point represents mean ± SEM from three independent replicates (*n* = 3). A detailed statistical evaluation is listed in Supplementary Table S7. **f** Detailed interactions between the N-terminus and other regions of the receptor are shown, with polar contacts depicted as blue dashed lines. The cryo-EM density maps for the N-terminal helix segment (from T15^Nter^ to R24^Nter^) and the linked disulfide bond (C25^Nter^–C278^7.25^) are depicted as gray surface. **g** Effects of mutations of key residues in the interface between N-terminus and other regions of receptor on the constitutive activity. The luminescence values achieved by vehicle treatment are normalized to those achieved by full activation or antagonism. Each data point represents mean ± SEM from three independent replicates (*n* = 3), each performed in triplicate. A detailed statistical evaluation is listed in Supplementary Table S8.

Despite the early identification of ATP and UTP as the endogenous ligands for the P2Y2R^7^, our findings also indicate that P2Y2R exhibits high constitutive G_q_ coupling in the absence of endogenous ligands (Supplementary Fig. S6a and Table S9). It is evident from a comprehensive investigation into G_q_ activation for P2Y1R-like receptors (Supplementary Fig. S6a). The G_q_ signaling activity of WT P2Y2R and mutants was assessed by a G_s/q_-based cAMP accumulation assay in HEK293 cells. To assess the constitutive activity of G_q_-coupled GPCRs, a novel chimeric G_q_ protein (G_s/q_) was engineered, enabling specific recognition of G_q_-coupled GPCRs and eliciting subsequent G_s_-dependent cAMP accumulation. P2Y2R, P2Y1R and P2Y6R exhibit relatively higher constitutive activity compared to P2Y4R, prostaglandin F2-alpha receptor (FP), and the Alpha-1A adrenergic receptor (α_1A_AR) with the latter two also as class A GPCRs (Supplementary Fig. S6a and Table S9). To elucidate the molecular mechanism of the high constitutive activity for P2Y2R, the structure of the P2Y2R in complex with G_q_ in the apo state was determined at a global resolution of 3.31 Å (Supplementary Fig. S9a–d). Remarkably, the map revealed a helix-like density at the N-terminus, situated within the orthosteric binding site (Fig. 4a). We modeled the N-terminal helix segment from T15^Nter^ to R24^Nter^, as well as the majority of the remaining receptor regions into the final refined map (Fig. 4f; Supplementary Fig. S9e). This N-terminal helix segment adopts a helical conformation and protrudes into the orthosteric ligand-binding pocket of the receptor in the apo state (Fig. 4b, d). The N-terminal helix-like segment inclines against TM6, TM7, and ECL3, positioning itself toward TM5 and ECL2, as observed in the overall structure (Fig. 4b). To assess the stability of the N-terminal segment anchored within the binding pocket of P2Y2R in its miniG_s/q_-coupled conformation, we performed three independent MD simulations on N-terminus-occupied P2Y2R–G_q_ complex, each with a timescale of 1 μs. Remarkably, at the end of each simulation, the N-terminal helix segment was still maintained, demonstrating the stability of this secondary structure (Supplementary Fig. S7a). The stable anchoring of the N-terminus is evident from the limited distance variations between residues W16^Nter^ and P189^ECL2^, compared with that observed in the cryo-EM structure, during three runs of MD simulations (Supplementary Fig. S7d).

**Fig. 5:**
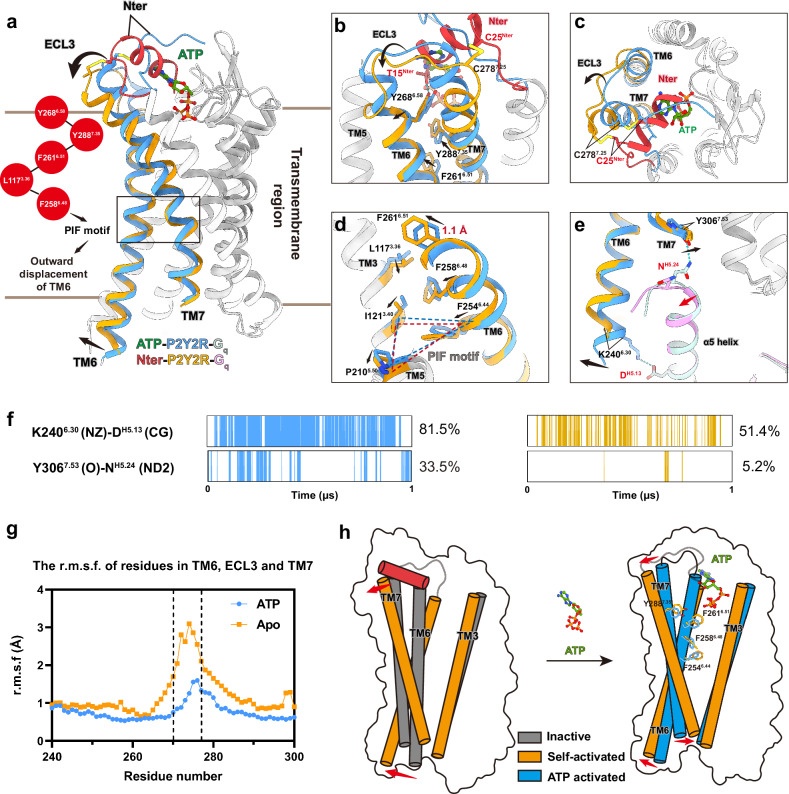
Different conformations of P2Y2R in the self-activated and fully activated states. **a** Structural superposition of the apo P2Y2R–G_q_ and ATP-bound P2Y2R–G_q_ complexes in side view. Receptors are aligned. For clarity, transmembrane helices TM6 and TM7 are highlighted in blue for the fully activated state and in orange for the self-activated state, while other TMs are colored in light gray. The movements of the TMs are indicated by black arrows, and key residues mediating conformational changes are displayed in the red circles. The binding pocket of the N-terminus (red) in the self-activated state is compared with that of ATP (green) in fully activated state. **b**, **c** Close-up representations of the N-terminus bound to the extracellular side of TM6 and TM7 in the self-activated state, compared with the ATP-bound fully activated state, are shown from the side (**b**) and top views (**c**). Key residues in P2Y2R mediating conformational changes are highlighted as sticks. The black arrows indicate the conformational changes of P2Y2R from the fully activated state to the self-activated state. Polar contacts are depicted as blue dashed lines. **d** Structure rearrangement of the conserved PIF motif in the self-activated state compared with the ATP-bound state. Key residues mediating the structure rearrangement are highlighted as sticks, and their conformational changes are indicated by black arrows. **e** Comparison of the P2Y2R–G_q_ interfaces in the self-activated state and the ATP-bound fully activated state. The movements of the α5 helix of the G_q_ and the TMs of the receptor are indicated by red and black arrows, respectively. Key residues displaying different interactions in these two states are highlighted as sticks. Polar contacts are depicted as blue dashed lines. **f** Comparison of two polar contacts of the receptor–G-protein interfaces in the ATP-bound P2Y2R–G_q_ complex and the N-terminus-occupied P2Y2R–G_q_ complex through MD simulations. One representative trace plot is displayed to illustrate this. The corresponding percentage values on the right represent the mean calculated from three independent simulations, each 3 µs in length. A detailed statistical evaluation for the data from three runs of MD simulation is listed in Supplementary Table S13. **g** The flexibility of TM6 and TM7 of P2Y2R in the self-activated state (labeled as ‘apo’) and the ATP-bound fully activated state (labeled as ‘ATP’) is quantified by MD simulations. The RMSF values of residues from K240^6.30^ to C300^7.47^ are shown, with the ECL3 region (residues S270–S277) highlighted by black dashed lines. **h** Schematic representation of the conformation of the P2Y2R in the AlphaFold2-predicted inactive, self-activated, and fully activated states. The conformational changes of transmembrane helices in the self-activated state relative to the inactive state and fully activated state are indicated by red arrows. Residues in TM6 and TM7 associated with activation are highlighted as sticks.

The structural superposition of the apo state P2Y2R–G_q_ complex and the ATP-bound P2Y2R–G_q_ complex demonstrates a partial overlap of the binding sites for the N-terminus and ATP (Fig. 5a–c). This observation prompted us to examine the potential role of the N-terminus in modulating the constitutive activity of P2Y2R. We first compared the constitutive activity of N-terminus truncated or swapped mutants of P2Y2R with its WT form through the G_s/q_-based cAMP accumulation assay. As expected, truncation of the N-terminus (P2Y2R_deltaN) or replacement that with P2Y4R N-terminus in P2Y2R (P2Y2R_Y4Nter) exhibits reduced constitutive activity, as indicated by the relative luminescence levels between the vehicle-treated group and the full agonist-treated group (Supplementary Fig. S6b–d and Tables S10, S11). However, truncation of the N-terminus had minimal impact on the constitutive activity of P2Y1R and P2Y6R (Supplementary Fig. S6b, e and Table S10), suggesting that the N-terminus is not responsible for the high constitutive activity of P2Y1R and P2Y6R. Further sequence analysis of P2Y2R reveals that this helix-like segment can be divided into three grouped residues separated by three glycine (Fig. 4d). To investigate the specific role of this helix-like segment of the N-terminus on the constitutive activity, the grouped residues in this helix-like segment are replaced by a GS linker to generate three mutants: swap1 (from T15^Nter^ to D17^Nter^), swap2 (from T15^Nter^ to L21^Nter^), and swap3 (from T15^Nter^ to R24^Nter^) (Fig. 4d). The agonist ATP and the potent selective antagonist AR-C 118925XX were utilized to fully activate or antagonize the P2Y2R, respectively^50^. The constitutive activities of each mutant were calculated by normalizing the luminescence values at vehicle treatment to those at the fully activated state (100%) and the fully antagonized state (0%). As shown by the dose-response curves of activation and antagonism, the swap1, swap2, and swap3 mutants exhibited 67%, 53%, and 12% constitutive activities relative to WT P2Y2R, corresponding to 85%, 93%, and 73% expression levels relative to WT P2Y2R (Fig. 4e; Supplementary Fig. S6h and Table S7). Furthermore, the N-terminus peptide from G14^Nter^ to R24^Nter^ was synthesized and demonstrated the ability to activate N-terminus-truncated P2Y2R (P2Y2R_Del_Nter) in a dose-dependent manner, confirming that this segment is able to activate the P2Y2R receptor either as part of the receptor at the N-terminus or as a separate peptide despite its lower activation efficacy relative to ATP (Supplementary Fig. S6f and Table S12). As expected, the N-terminus peptide can antagonize the ATP-induced G_q_ activation of P2Y2R_Del_Nter (Supplementary Fig. S6g and Table S12).

The helix-like segment, featuring multiple negatively charged amino acids, forms extensive interactions with TM5 and TM6, as well as ECL2 and ECL3 in P2Y2R (Fig. 4f). The conformation of the N-terminus is restricted by a disulfide bond between C25^Nter^ and C278^7.25^. Breaking the formation of disulfide bond through alanine mutation of either C25^Nter^ or C278^7.25^ almost entirely abolished the constitutive activity of the receptor (Fig. 4g; Supplementary Table S8). Inspection of the structural interactions reveals the key residues that contribute to the self-activation of the N-terminus. Specifically, T15^Nter^ deeply inserts into the binding pocket and forms a hydrogen bond with the side chain of Y268^6.58^ in TM6 and D185^ECL2^ in ECL2 (Fig. 4f). Furthermore, D17^Nter^ forms a salt bridge interaction with R272^6.62^, which causes R272^6.62^ to protrude from TM6 in an unwound conformation (Fig. 4f). It is worth emphasizing that Y268^6.58^ and R272^6.62^ play crucial roles in ATP recognition (Fig. 2a; Supplementary Fig. S5b, e, j). Extensive hydrophobic interactions are also formed between the N-terminus and other regions of the receptor. For instance, L21^Nter^ and W16^Nter^ are engaged in hydrophobic interactions with the receptor, where L21^Nter^ forms a hydrophobic contact with L276^ECL3^ and W16^Nter^ is snugly embedded within a hydrophobic pocket formed by residues H184^ECL2^, P189^ECL2^, and F192^5.32^ (Fig. 4f). Consistent with the structural observations, alanine mutations of H184^ECL2^, D185^ECL2^, P189^ECL2^, F192^5.32^, Y268^6.58^, R272^6.62^, and L276^ECL3^ significantly impaired the constitutive activity of P2Y2R (Fig. 4g; Supplementary Table S8). Therefore, the helix-like segment in the N-terminus serves as a ‘built-in agonist’ by occupying the ligand-binding pocket, facilitating self-activation and thereby maintaining the constitutive activity of P2Y2R (Fig. 4c).

The distinctive N-terminus conformation and its involvement in P2Y2R’s self-activation bear striking resemblance to the orphan GPCR GPR20. GPR20 also possesses an N-terminus that occupies the orthosteric pocket and is associated with self-activation^51^. Comparison of P2Y2R with GPR20 reveals a similar N-terminus conformation but different interaction modes (Supplementary Fig. S7f). Specifically, the helix-like segment of the N-terminus in P2Y2R occupies a relatively superficial location, positioning it closer to TM6 and TM7 compared to its counterpart in GPR20 (Supplementary Fig. S7f). In the case of other orphan GPCRs, such as GPR12, GPR17, GPR21, GPR52, and GPR161, it is ECL2 rather than the N-terminus that functions as a crucial determinant of their inherent constitutive activity^52–57^. Structural comparison highlights that the N-terminus of P2Y2R exhibits shallower penetration into the binding cavity compared to ECL2 in these orphan GPCRs (Supplementary Fig. S7g). Similarly, the disulfide bond between ECL2 and TM3 contributes to stabilizing the ECL2 conformation in some of these receptors, such as GPR17, GPR21, GPR52, and GPR161, which is associated with their constitutive activity^53–56^. Interestingly, phylogenetic analysis indicates that the specific sequence features of the P2Y2R N-terminus are conserved across mammalian species (Supplementary Fig. S7e), suggesting that the self-activation mechanism in P2Y2R may contribute to the complex physiological regulation of many mammalian systems.

### Comparison between self-activation state and fully activated state of P2Y2R

Occupancy of the orthosteric binding site by the P2Y2R N-terminus makes the receptor adopt structural configurations indicative of an active state. Similar to ATP-induced full activation (Supplementary Fig. S1b, d), the outward displacement of TM6 and the packing of residues R131^3.50^, Y218^5.58^, and Y306^7.53^ (Supplementary Fig. S7b, c) collectively contribute to the creation of a binding cavity on the intracellular face for accommodating the α5 helix of G_q_. Structural overlays highlight the conformational differences between the N-terminus-occupied self-activation state and the ATP-bound active state (Fig. 5a). This observation prompted an investigation into the influence of the N-terminus on the structural arrangements related to self-activation. In the self-activated state, the extracellular ends of TM6, TM7, and ECL3 in P2Y2R display notable outward displacement compared to the ATP-bound state (Fig. 5a–c). More specifically, the extracellular end of TM6 unwinds into a short loop (Fig. 5b, c). Collectively, our data reveal enhanced flexibility at the extracellular ends of TM6 and TM7, as well as ECL3 in P2Y2R’s self-activation state compared to the fully activated state, as indicated by a significant increase in the root mean square fluctuation (RMSF) values computed from MD simulations (Fig. 5g). These conformational changes are mediated by interactions between the N-terminus and the transmembrane core (Fig. 5b). Specifically, residue T15^Nter^ forms a hydrogen bond with Y268^6.58^, which stabilizes Y268^6.58^ in an outward-shifted position. It should be noted that the aromatic residues Y288^7.35^, F261^6.51^ and F258^6.48^ form a hydrophobic interaction core associated with activation (Supplementary Fig. S1a, c). The displacement of Y268^6.58^ facilitates the structural rearrangements of these aromatic residues (Fig. 5b, d, h), leading to an ~1.1 Å shift towards TM3 of the residue F261^6.51^ and a flip of the toggle switch residue F258^6.48^ (Fig. 5d). The altered conformation of F258^6.48^ further influences the conserved P210^5.50^-I121^3.40^-F254^6.44^ motif, which in turn mediates conformational adjustments within the intracellular domains of TM5 and TM6 (Fig. 5d, e). Finally, the intracellular end of TM6 exhibits a more pronounced outward displacement in the self-activated state compared to the ATP-activated state (Fig. 5e, h).

Although the self-activated and ATP-activated conformations of the P2Y2R generally share similarities in their interactions with G_q_ (Supplementary Table S15), distinct interactions between the receptor and G_q_ can be observed due to the structural arrangements of TM6 and TM7 (Fig. 5e, h). Due to the outward displacement of TM6, the salt bridge between K240^6.30^ and D^H5.13^ of G_q_ is impaired in the self-activated state compared with the ATP-bound fully activated state (Fig. 5e). Furthermore, with the slight movement of the α5 helix of G_q_ towards TM5 and TM6, the N^H5.24^ from the ‘wavy hook’ loses its hydrogen bond interaction with the backbone carbonyl of Y306^7.53^ in TM7 in the self-activated state, leading to different conformations of the intracellular end of TM7 (Fig. 5e). These different interactions between the self-activated and ATP-activated states are further supported by MD simulations (Fig. 5f; Supplementary Table S13). The polar contacts for pairs K240^6.30^–D^H5.13^ and Y306^7.53^–N^H5.24^ in the self-activated P2Y2R exhibit weaker interactions relative to their counterparts in the ATP-activated form. Therefore, the rearrangement of the hydrophobic interaction core in TM6 and TM7 subsequently contributes to the conformational changes on the intracellular side of the receptor and tunes the P2Y2R–G_q_ protein interactions (Fig. 5h). These weaker interactions may explain the relatively lower magnitude of self-activation compared with ATP-induced full activation.

## Discussion

The metabotropic purinergic P2YRs, comprising P2Y1R-like and P2Y12R-like subfamilies, play a crucial role in recognizing nucleotides across various mammalian species^58^. The existence of multiple nucleotides recognized by different P2Y subtypes contributes to the complexity and nuance of purinergic signaling^30^. In this study, we report the cryo-EM structures of P2Y2R in complex with G_q_ or G_o_, in both ATP-bound and apo states, and UTP-bound P2Y4R in complex with G_q_. Combining structural analysis, functional assays, and MD simulations, we elucidate the activation mechanism of P2Y2R, revealing key respects of nucleotide recognition, G-protein subtype selectivity, and N-terminus-induced self-activation. These findings collectively advance our understanding of P2Y2R activation and lay groundwork for structure-based drug design.

The structure comparison and sequence analysis reveal distinct nucleotide-binding pockets in P2Y1R-like and P2Y12R-like receptors, indicating subtype-specific differences in ligand recognition and binding. In P2Y1R, P2Y2R, and P2Y4R, nucleotides bind in a superficial pocket, adopting a ‘base-up’ orientation. In contrast, P2Y12R binds nucleotides in a deeper and more secluded pocket with a ‘base-down’ orientation. The ligand-binding pockets of P2Y2R and P2Y4R, featuring a conserved cluster of positively charged residues from transmembrane helix core, specifically recognize the triphosphate moieties of ATP and UTP. Although P2Y2R and P2Y4R share considerable sequence homology and great similarities in ligand binding mode, differences in their N-terminal sequence likely contribute to their distinct ligand recognition specificities. The N-terminus in both P2Y2R and P2Y4R projects into the transmembrane helix bundle, forming extensive interactions with the base moiety of different nucleotides and modulating ligand recognition and specificity. The distinct sub-pocket architecture formed by the N-terminus in P2Y2R and P2Y4R underlies the differential agonistic properties of ATP, acting as a full agonist of P2Y2R but only a partial agonist of P2Y4R. Indeed, some nucleotide derivatives with increased P2Y2R selectivity have been successfully developed, even in the absence of experimentally determined P2Y2R structures. For example, 2-thio-UTP has been described as a P2Y2R agonist with 10-fold selectivity over P2Y4R^38,59^. With the high-resolution cryo-EM structures of P2Y2R and P2Y4R determined in this study, it is expected that structure-based rational design can be further carried out to develop compounds with better P2Y2R subtype selectivity over P2Y4R through nucleobase modifications of UTP and ATP.

Prior research has elucidated a unique signaling pathway for P2Y2R activation, wherein integrin serves as a key mediator^16,17^. In this pathway, P2Y2R interacts with α_V_β_3/5_ through an Arg-Gly-Asp (RGD) motif in its ECL1, eliciting downstream signaling mediated by the heterotrimeric G_o_ and G_12_ proteins, which results in cytoskeletal rearrangements associated with chemotaxis and neurite extension^16,44^. Given that G_o_ is the most abundant G-protein subtype in the brain^60^, the P2Y2R–G_o_ signaling pathway is critical for maintaining cellular homeostasis within the central nervous system, underscoring its therapeutical potential. Our comprehensive analysis of G-protein coupling demonstrates that the P2Y2R exhibits promiscuous G-protein coupling, concurrently interacting with both G_q_ and G_o_ proteins in response to ATP. As revealed by the P2Y2R–G-protein complex structure, binding of ATP facilitates the activation of P2Y2R for coupling both G-proteins without engagement to α_V_β_3/5_. Through integrated structural and functional analysis, we reveal the molecular basis for P2Y2R’s differential abilities of coupling with G_q_ and G_o_ via structural variations at the receptor–G-protein interfaces. Notably, our findings show that the ICL2 plays a more pronounced role in mediating G_q_ coupling, whereas the ICL3 plays a dominant role in facilitating G_o_ coupling. Accordingly, specific mutations in the ICL2 and ICL3 of P2Y2R differentially impair G_q_ and G_o_ signaling pathways, as shown by our functional assays. These mutants can thereby be used to explore the physiological functions of specific G-protein pathways of P2Y2R in mechanistic studies. Furthermore, ICL2 and ICL3 of P2Y2R may present promising allosteric sites for the development of allosteric modulators with G-protein subtype selectivity. In agreement with our studies, a similar role of ICL3 in determining G_i/o_ selectivity was observed in other reported GPCRs, such as the Galanin Receptor 1 (GALR1) and Somatostatin Receptor 2 (SSTR2)^45,46^. However, in other cases, the ICL3 of the Cholecystokinin A Receptor (CCK(A)R) and Protease-Activated Receptor 1 (PAR1) plays a more crucial role in G_q_ coupling than in G_i_ coupling^61,62^. These investigations reveal the complexity of ICL3 in dictating the specificity of receptor coupling with distinct G-protein subtypes.

Compared to most class A GPCRs, P2Y2R displays an unusually high level of constitutive activity in the absence of its endogenous agonist ATP. Our structural investigation of apo-P2Y2R–G_q_ complex uncovers a unique P2Y2R activation mechanism, wherein the N-terminus occupies the orthosteric ligand-binding pocket and functions as a ‘built-in’ agonist to induce self-activation. This result provides another rational explanation for the physiologically relevant constitutive activity of P2Y2R as observed in previous studies and confirmed in our study^18–20^. Indeed, ‘built-in’ structural elements such as the N-terminus and ECL2 have been observed to play a vital role in the self-activation of many orphan GPCRs^51–57^. For example, GPR17, an orphan GPCR that is phylogenetically related to the P2YR, can be self-activated by its ECL2^53^. Through our functional assays in P2YRs, we observed significant basal activities in P2Y1R and P2Y6R even in the absence of agonists, suggesting that constitutive activity is a common feature among P2Y1R-like receptors. Unlike other N-terminus self-activated GPCRs, such as GPR20^51^, the N-terminus of P2Y2R adopts a relatively shallow position, anchored by a stabilizing disulfide bond between C25^Nter^ and C278^7.25^. The interplay between the N-terminus, TM6, and ECL3 gives rise to the structural arrangements of TM6 and TM7, which plays a pivotal role in facilitating P2Y2R activation. Therefore, the identification of a previously unrecognized binding pocket for the N-terminus presents a promising opportunity for designing peptide therapeutics that modulate P2Y2R activity, offering potential treatment for chronic inflammatory diseases, cancer, and other conditions^15^. Beyond these intrinsic structural elements, the self-activation of GPCRs can also be influenced by other inherent factors. Notably, endogenous lipids have been reported to contribute to high constitutive activity for some orphan GPCRs, such as GPR174 and GPR161^56,57^. In GPR174, the endogenous lysoPS located on the surface of TM3, TM4, and TM5 is related to its constitutive activation^57^. In GPR161, a sterol located between TM6 and TM7 is required for constitutive G_s_ coupling^56^. Interestingly, electron density maps of P2Y2R also reveal some sterol-like molecules within the transmembrane domain, specifically in the cleft formed by TM3, TM4, and TM5 or TM2, TM3, and TM4. These embedded endogenous lipids may contribute to the constitutive activity of P2Y2R, although further investigation is needed to confirm their role.

Our studies on the constitutive activity of the P2Y2R raise questions about the physiological function of self-activation in vivo. It appears that purinergic signaling by the P2Y2R is not only regulated by the endogenous release of ATP under certain physiological conditions but also by its constitutive activity, which is related to the expression level on the cell membrane. Therefore, regulating the expression level of the P2Y2R strongly influence purinergic signaling. Notably, the dynamic expression pattern of the P2Y2R can be detected, especially in response to cellular stimuli such as tissue injure and inflammation^63–65^. Specifically, P2Y2R mRNA levels were upregulated in glial cells of the cortex and nucleus accumbens in rats during trauma, promoting astrocyte survival after injury and neuroprotection^66,67^. It is suggested that the modulation of the constitutive activity of P2Y2R is physiologically relevant. Interestingly, the specific sequence features of the P2Y2R N-terminus appear to have evolved in mammals, as revealed by phylogenetic analysis. Emerging studies suggest that the P2Y2R plays a very important role in nociception^68,69^. Additionally, P2Y2R activation can regulate the expression of acetylcholinesterase and acetylcholine receptors at neuromuscular junctions in vertebrate^70^. We hypothesize that the self-activation of the P2Y2R may contribute to the complex physiological regulation in mammalian animals. More comparative studies are needed to elucidate the evolution and specific functions of the constitutive activity of the P2Y2R in mammals.

## Materials and methods

### Construct design and protein expression

The full-length WT human P2Y2R and P2Y4R genes were subcloned into a pcDNA3.1(–) vector for expression. The constructs included an N-terminal hemagglutinin (HA) signal peptide sequence, followed by a FLAG tag (DYKDDDDA). Engineered mini-G_s/q_ and mini-G_o_ proteins were fused to the C-termini of the receptors, respectively, with a flexible glycine/serine linker (GGSGG) and a 3C protease recognition site (LEVLFQGP) preceding the fusion. This design was chosen based on the established utility of mini-G protein fusions and G-protein chimera strategies in stabilizing and elucidating the active state structures of GPCRs^71,72^. To enhance the binding affinity for subsequent studies, the N-terminal sequence of G_i1_ was incorporated into the mini-G protein, which also facilitated the interaction with scFv16. In addition, a D320^7.49^N mutation, previously reported to improve the stability and expression of P2Y1R, was introduced into the P2Y2R construct^24^. The plasmids encoding the fusion proteins were transiently transfected into Expi293F cells (Thermo Fisher Scientific) at a cell density of 2 ×10^6^ cells/mL using polyethylenimine (PEI, Polysciences) as the transfection reagent. Post transfection, the cells were cultured in SMM293-TII medium (Sino Biological) supplemented with 10% fetal bovine serum (FBS) and incubated under conditions of shaking at 130 rpm, 37 °C, and 8% CO_2_ for 48 h. Following this incubation period, the cells were harvested by centrifugation at 4000 rpm for 20 min and stored at –80 °C for subsequent protein purification.

### Purification and reconstitution of P2Y2R–G_o_/G_q_ and P2Y4R–G_q_ complexes

For purification of the P2Y2R–miniG_q_/miniG_o_, cell pellets were thawed and lysed in a buffer containing 20 mM HEPES-NaOH (pH 7.5), 2 mM EDTA, and protease inhibitors (2.5 μg/mL leupeptin and 160 μg/mL benzamidine). The lysate was centrifuged at 18,000 rpm for 20 min to isolate the membrane fraction, followed by a wash with a hypertonic buffer (20 mM HEPES-NaOH, pH 7.5, 750 mM NaCl, 2 mM MgCl_2_, and protease inhibitors). Prior to solubilization, the membrane fractions were resuspended in a binding buffer (20 mM HEPES-NaOH, pH 7.5, 100 mM NaCl, 2 mM MgCl_2_, supplemented with protease inhibitors, benzonase nuclease, 2 mg/mL iodoacetamide, and 1 mM ATP) and incubated overnight at 4 °C to facilitate the formation of the receptor–G protein complex. On the second day, the membrane fractions were homogenized using a Dounce homogenizer in a solubilization buffer (20 mM HEPES-NaOH, pH 7.5, 500 mM NaCl, 2 mM MgCl_2_, 2 mM CaCl_2_, 30% (v/v) glycerol, 1% (w/v) *n*-dodecyl-β-D-maltopyranoside (DDM, Anatrace), 0.1% (w/v) cholesteryl hemisuccinate (CHS, Sigma), protease inhibitors, 2 mg/mL iodoacetamide, and 1 mM ATP). After solubilization for 2 h at 4 °C, the supernatant was isolated by centrifugation and applied to an M1 anti-FLAG affinity column. For detergent exchange, DDM was exchanged to lauryl maltose neopentyl glycol (LMNG, Anatrace) on the M1-FLAG column, with the salt concentration gradually reduced to 100 mM. Target protein was eluted with an elution buffer (20 mM HEPES-NaOH, pH 7.5, 100 mM NaCl, 10% (v/v) glycerol, 0.005% LMNG, 0.0005% CHS, 2 mM EDTA, 250 μg/mL FLAG peptide, and 1 mM ATP). The receptor was concentrated using a 50 kDa molecular weight cutoff spin concentrator (Millipore) to the desired volume for further use.

To reconstitute the receptor–G-protein complex, Gβγ, Nb35 and scFv16 were expressed and purified following previously described methods^71,73^. For the ATP-bound P2Y2R–G_q_/G_o_ complex, P2Y2R–miniG_q_ (or P2Y2R–miniG_o_), Gβγ, and Nb35 (or scFv16) were mixed in a 1:1.2:1.2 molar ratio and supplemented with 2 mM MgCl_2_. After 1 h of incubation at 4 °C, the heterotrimeric complex was purified by size-exclusion chromatography using a Superdex S200 Increase 10/300 GL column (GE Healthcare) equilibrated with a buffer containing 20 mM HEPES-NaOH (pH 7.5), 100 mM NaCl, 0.002% (w/v) LMNG, 0.0002% CHS, 2 mM MgCl_2_, and 1 mM ATP. For the apo P2Y2R–G_q_ complex, the protein was purified and assembled as described, without adding any ligands. In the case of the UTP-bound P2Y4R–G_q_ complex, the purification and assembly process followed that of the P2Y2R–G_q_ complex, with the exception that 1 mM UTP was used instead. The resulting heterotrimeric complexes were then concentrated to 4–8 mg/mL for making cryo-EM samples.

### Cryo-EM sample preparation and data collection

The Au200 girds (QUANTIFOIL R 1.2/1.3) were treated under vacuum for 2 min and then subjected to glow discharge for 35 s. For sample preparation, 4 μL of the protein solution was applied onto each glow-discharged grid. After that, the grids were blotted for 4.5 s with a blotting force of 0 using a FEI Vitrobot Mark IV (Thermo Fisher Scientific) and subsequently plunged into liquid ethane. These procedures were conducted at a constant temperature of 8 °C and 100% humidity. After checking the quality of the protein samples on the grids, micrographs were collected using a Titan Krios (Thermo Fisher Scientific) electron microscope equipped with a Gatan K3 Summit detector and a GIF quantum energy filter with a 20 eV slit width. Images were recorded at a magnification of 64,000×, with each movie stack consisting of 32 frames over an exposure time of 2.6 s, resulting in a total dose of 50 e^–^/Å². The defocus values for each image were set from 1.1 μm to 1.6 μm. Data acquisition was automated using EPU software (Thermo Fisher Scientific). All frames in each stack were aligned and summed using the MotionCor2 and binned to a pixel size of 1.08 Å^74^.

### Cryo-EM data processing

A total of 1403, 1072, 804, and 904 micrographs were collected for ATP–P2Y2R–miniG_q_–Gβγ–Nb35, ATP–P2Y2R–miniG_o_–Gβγ–scFv16, apo-P2Y2R–miniG_q_–Gβγ–Nb35, and UTP–P2Y4R–miniG_q_–Gβγ–Nb35, respectively. All movie stacks were motion-corrected using MotionCor2 before being imported into cryoSPARC 3.2 and 4.1 for further processing^75^. Contrast transfer function (CTF) parameters were estimated using Patch CTF Estimation in cryoSPARC, and micrographs with a CTF fit resolution better than 4 Å were selected for subsequent analysis. Particles were initially picked using the blob picker, followed by extraction in a 228-pixel box (Fourier cropped to 128 pixels). Two-dimensional (2D) class averages with clear structural features were used as templates for additional particle picking. Particles from template picking were subjected to 2D classification and ab-initio reconstruction to generate an initial reference volume. Heterogeneous refinement was performed with three classes, including one good reference volume and two bad volumes reconstructed from poor 2D classes. After multiple rounds of heterogeneous refinement, inferior particles were progressively separated from high-quality particles. Additional extraction without binning and further heterogeneous refinement were performed to yield particles suitable for nonuniform refinement and local refinement, resulting in high-resolution maps. Data collection and processing parameters for all structures are summarized in Supplementary Table S16.

### Model building and refinement

Initial structural models of P2Y2R and P2Y4R were predicted from AlphaFold2^76^. The structures of the receptors were combined with miniG_s/q_–Gβγ–Nb35 from NK1R–G_q_ complex (PDB: 7RMI)^71^ or miniG_o_–Gβγ–scFv16 from hM4Di–G_o_ complex (PDB: 8E9X)^77^ in PyMOL (Schrödinger). Subsequently, the generated full complex structure models were initially fitted into the density map using UCSF Chimera X^78^. All the models were manually adjusted by COOT 0.9-pre^79^ and further refined with Phenix^80^ using secondary structure restraints. The models were finally validated using Phenix and MolProbity^81^. Refinement parameters for all structures are summarized in Supplementary Table S16. Structure figures were all prepared using UCSF Chimera X.

### NanoBiT-based G-protein dissociation assay

Different G-protein subtype couplings of P2Y2R were monitored using the G-protein dissociation assay^82^. HEK293T cells (Thermo Fisher Scientific) were seeded in a 6-well plate on the first day. On the second day, when the cells reached 70%–80% confluency, plasmids encoding P2Y2R (500 ng), Gα-LgBiT (1 µg), Gβ_1_ (500 ng), Gγ_2_-SmBiT (500 ng), and Ric8A (500 ng, used in Gα_q_, Gα_12_, and Gα_13_) were co-transfected into the HEK293T cells using PEI (Polysciences). The following Gα-LgBiT constructs were used: Gα_q_-LgBiT, Gα_12_-LgBiT, Gα_13_-LgBiT, Gα_o_-LgBiT, Gα_i1_-LgBiT, Gα_i2_-LgBiT, and Gα_i3_-LgBiT. On the third day, the transfected HEK293T cells were seeded into 96-well plates. After one day of recovery, the cells were washed once with Hanks’ Balanced Salt Solution (HBSS) buffer and then incubated in a reaction buffer containing HBSS supplemented with 10 µM coelenterazine at room temperature for 1 h. Relative luminescence units (RLUs) were measured in 5 min after adding the P2Y2R agonist using a PerkinElmer plate reader. The obtained luminescence values in response to agonist stimulation were normalized to the baseline values. The normalized values from three independent experiments were plotted as a function of agonist concentration using nonlinear regression of one-phase exponential decay in GraphPad Prism 9.0.

### cAMP accumulation assay

A chimeric G-protein (G_s/q_) was designed to recognize G_q_-coupled GPCRs but exhibit G_s_-dependent cAMP generation based on the mini-G_s/q_70 design^72^. The cAMP accumulation level was used to assess the constitutive activity of G_q_-coupled GPCRs. For the assay, plasmids encoding the GPCR, G_s/q_, and pGloSensor-22F cAMP (Promega) were co-transfected into HEK293T cells at a ratio of 1:1:40 in a 6-well plate using PEI (Polysciences) when the cells reached 70%–80% confluency. One day post transfection, the cells were seeded into 96-well plates. On the next day, the cells were washed once and incubated in HBSS buffer supplemented with 2% GloSensor reagent (Promega). The cells were then incubated at 37 °C in a CO_2_ incubator for 1 h, followed by an additional 1-h incubation at room temperature. RLUs were measured using a PerkinElmer plate reader under agonist treatment, inverse agonist treatment, or buffer control conditions. For the constitutive evaluation of P2Y2R, the luminescence values obtained from vehicle treatment were normalized to those from full activation or antagonism, calculated by the following formula: constitutive activity = (RLU_vehicle_ – RLU_inverse agonist_)/(RLU_agonist_ – RLU_inverse agonist_). For comparison of different GPCRs (Supplementary Fig. S6a), the constitutive activity was determined using the following formula: constitutive activity = (RLU_vehicle_ – RLU_CTL_)/(RLU_agonist_ – RLU_CTL_). RLU_CTL_ was obtained by the same protocol but using control cells transfected with G_s/q_ and pGloSensor-22F plasmids without the receptor plasmid.

### Expression detection of different constructs

Expression levels of different GPCRs in this study (Supplementary Fig. S6a) were measured using cell surface staining. In brief, HEK293T cells were transfected with pcDNA3.1(–) plasmids containing GPCRs with an N-terminal HA sequence followed by a FLAG tag. One day post transfection, the cells were resuspended and incubated in HBSS buffer supplemented with 1 mM CaCl_2_ and 50 nM FITC-conjugated M1-FLAG antibody for 15 min, followed by a wash with HBSS buffer containing CaCl_2_. Expression levels were analyzed by flow cytometry (BD Accuri™ C6 Plus). Approximately 10,000 events were recorded for each sample. The FITC signal was detected by flow cytometry to reflect the expression level, with excitation at 488 nm and emission at 519 nm. Each measurement was performed in triplicate. Data were processed using FlowJo version 10.

Expression levels of WT P2Y2R, P2Y4R, and their corresponding mutants were measured using the HiBiT assay^83^. The expression constructs included an N-terminal HA sequence, followed by a FLAG tag and a HiBiT tag (VSGWRLFKKIS). After transfection in HEK293T cells, the cells were seeded into 96-well plates. On the next day, the cells were incubated in a reaction buffer containing HBSS supplemented with 10 µM coelenterazine at room temperature for 1 h. RLUs were measured using a PerkinElmer plate reader 10 min after adding 1 µM purified LgBiT protein. Each measurement was performed in triplicate. RLUs indicate the expression level of the receptors.

### MD simulations

All simulations were performed by using the CHARMM36m parameter set (with CMAP protein backbone energy correction terms) for proteins, lipids, ATP, salt ions, and the CHARMM TIP3P water model. ATP–P2Y2R–G_q_, ATP–P2Y2R–G_o,_ and apo-P2Y2R–G_q_ complex structures were simulated. Hydrogen atoms were added using Maestro (Schrodinger LLC, New York). Prime (Schrodinger) was used to model the missing sidechains and the missing ICL3. All nonprotein molecules were removed except ATP and the crystallographic water molecules. The nanobody was also removed. The protein chain termini were capped with an acetyl group at the N-terminus and a methylamino group at the C-terminus. All Lys and Arg residues were protonated and all His, Glu, and Asp residues were deprotonated. All simulations were performed on a single graphical processing unit using Amber22 Compute Unified Device Architecture version of particle-mesh Ewald MD^84,85^. Prepared protein structures were inserted into a large equilibrated POPC (1-palmitoyl-2-oleoyl-sn-glycero-3-phosphocholine) bilayer solvated with 0.15 M NaCl. Heating (to 310 K over 150 ps) and equilibration (50 ns with restraints on protein and ligand) steps were performed before production simulations. Production simulations were performed in the NPT ensemble at 310 K and 1 bar, using a Langevin thermostat for temperature coupling and a Monte Carlo barostat for pressure coupling. Bond lengths to hydrogen atoms were constrained using SHAKE^86^. Non-bonded interactions were cut off at 12 Å. Trajectory snapshots were saved every 200 ps. All simulations were 1 μs in length. Three independent simulations were performed for each complex. The CPPTRAJ package in AmberTools23 was used for post-analysis^87,88^. Visual Molecular Dynamics was used to visualize the trajectories^89^. Time traces from the simulations were smoothed using a moving average with a window size of 2 ns.

## Supplementary information

Supplementary information

## Data Availability

All structures in this study have been deposited in the Protein Data Bank (PDB) under the accession codes 9K0X, 9K20, 9K25 and 9K0K. The EM maps have been deposited at the Electron Microscopy Data Bank (EMDB) under the accession numbers EMD-61958, EMD-61986, EMD-61990, and EMD-61947.
