# Supplementary material for: Structural insight into the self-activation and G-protein coupling of P2Y2 receptor
Source: Cell Discov. 2025 May 13;11:47. doi: 10.1038/s41421-025-00797-x (PMC12075631; doi:10.1038/s41421-025-00797-x)
Supplement: Supplementary file 1 — Supplementary information [file 41421_2025_797_MOESM1_ESM.pdf]

Supplementary Information

Supplementary Figs. S1-S10

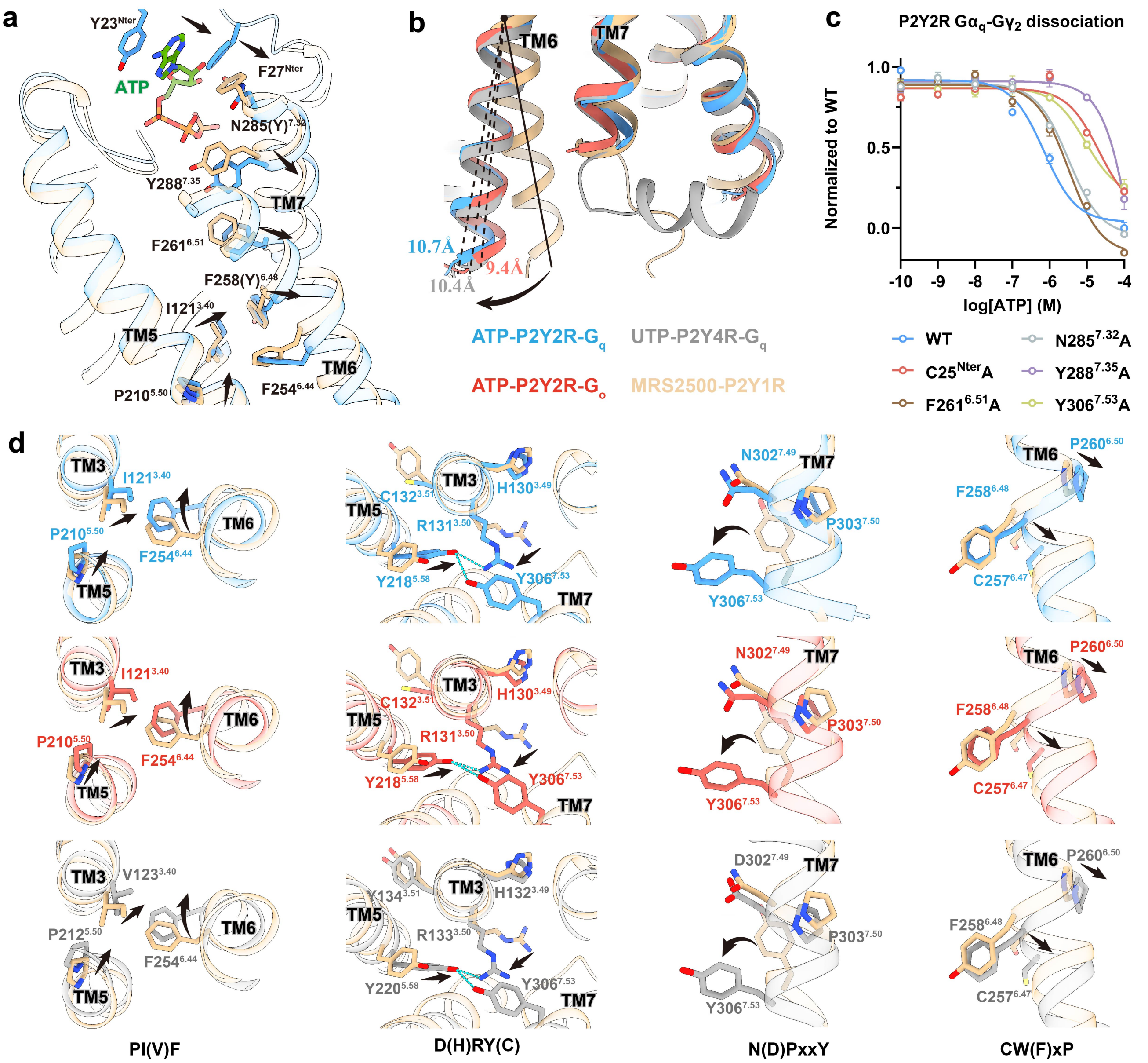

Supplementary Fig. S1 Activation hallmarks of P2Y2R and P2Y4R. a Comparison between the active structures of P2Y2R and the inactive structure of P2Y1R (PDB ID: 4XNW). The residues in TM3, TM5, TM6 and TM7 associated with P2Y2R activation are shown as sticks, and the activation pathway is indicated by the conformational changes of these residues, which are displayed by black arrows. **b** Structural superposition of the active structures of P2Y2R and P2Y4R with the inactive structure of P2Y1R (PDB ID: 4XNW). The receptors are aligned. The black arrows indicate the displacement of TM6 on the intracellular side. **c** Effects of

mutations of key residues in the activation pathway of P2Y2R on $G\alpha_q$-$G\gamma_2$ dissociation induced by ATP. The dose-response curves represent the global fit of mean ± s.e.m. from grouped data in three independent experimental replicates (n=3). A detailed statistical evaluation is listed in Supplementary Table S3. **d** Close-up views of the $P^{5.50}I(V)^{3.40}F^{6.44}$, $D(H)R^{3.50}Y(C)$, $CW(F)^{6.48}xP$ and $N(D)PxxY^{7.53}$ motifs displaying conformational changes on receptor activation. The conformational changes of these motifs are indicated by black arrows. Polar contacts are depicted as blue dashed lines.

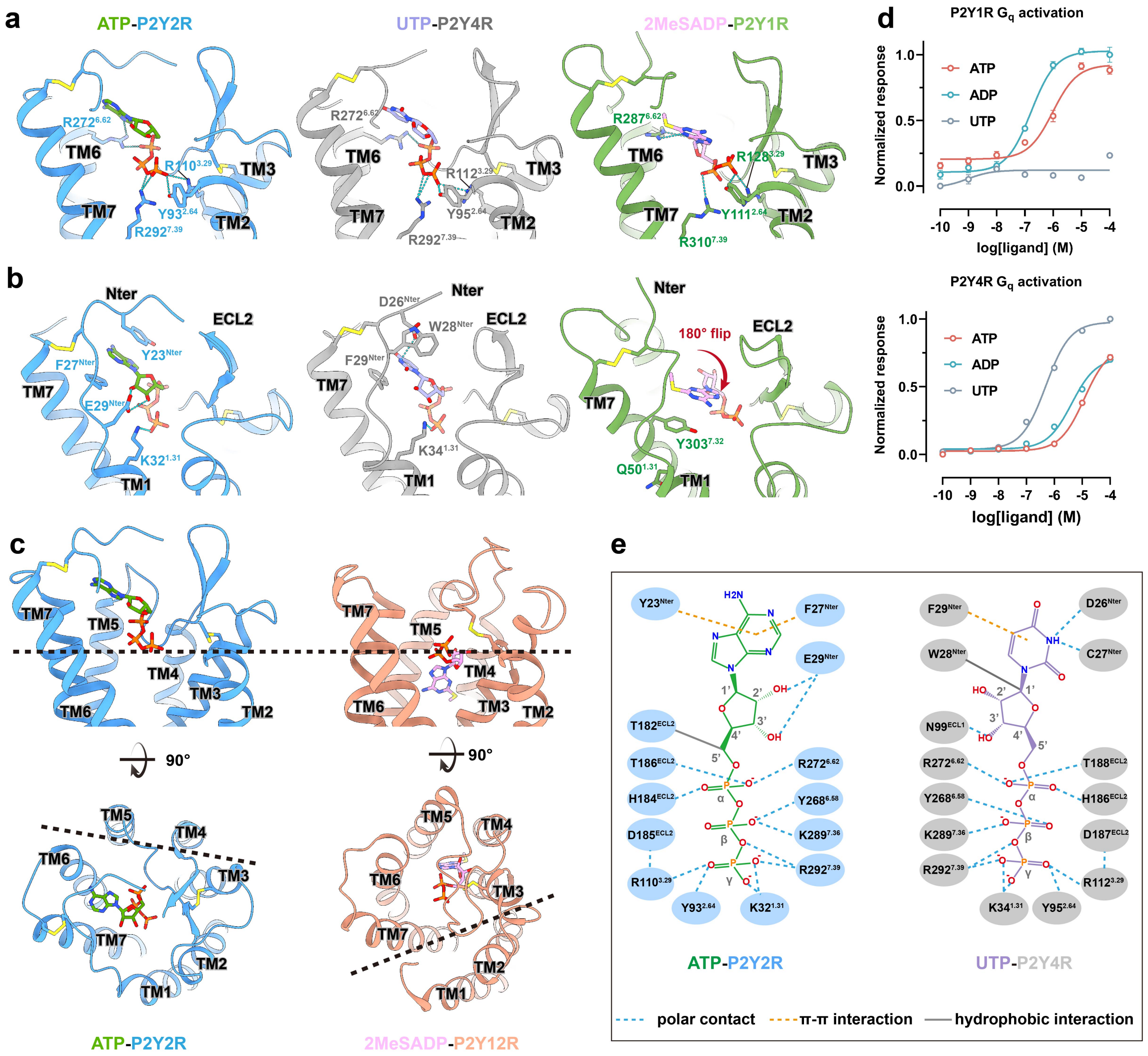

Supplementary Fig. S2 Comparison of the ligand binding pocket of P2Y1R, P2Y2R, P2Y4R, and P2Y12R. **a, b** Conserved (**a**) and specific (**b**) recognition of ATP, UTP, and 2MeSADP by P2Y2R, P2Y4R and P2Y1R (PDB ID: 7XXH). The interaction residues are highlighted as sticks in the same colors as the cartoon representation. Disulfide bonds are depicted as yellow sticks, and polar contacts are depicted as blue dashed lines. **c** Comparison of the ligand binding pocket between the P2Y2R and P2Y12R (PDB ID: 7XXI) from top and side view. Black dashed lines across the transmembrane core indicate the differences of ligand binding pose between P2Y2R and P2Y12R. **d** Dose-response curves for P2Y1R and P2Y4R in response to different ligands determined by $G_{s/q}$-based cAMP accumulation assay. The Delta (Δ) luminescence values before and after agonist treatment are used to reflect receptor activation and are normalized to the fraction of response under treatment

with ADP for P2Y1R and UTP for P2Y4R, respectively. The dose-response curves represent the global fit of mean ± s.e.m. from grouped data in three independent experimental replicates (n=3). A detailed statistical evaluation is listed in Supplementary Table S1. **e** Schematic representation of the ATP and UTP recognized by the P2Y2R and P2Y4R. Polar contacts, including hydrogen bonds and salt bridges, are indicated by light blue dashed lines, and π-π interactions are indicated by orange dashed lines. Hydrophobic interactions are indicated as grey solid lines. Chemical structures of the ATP and UTP are shown and labeled.

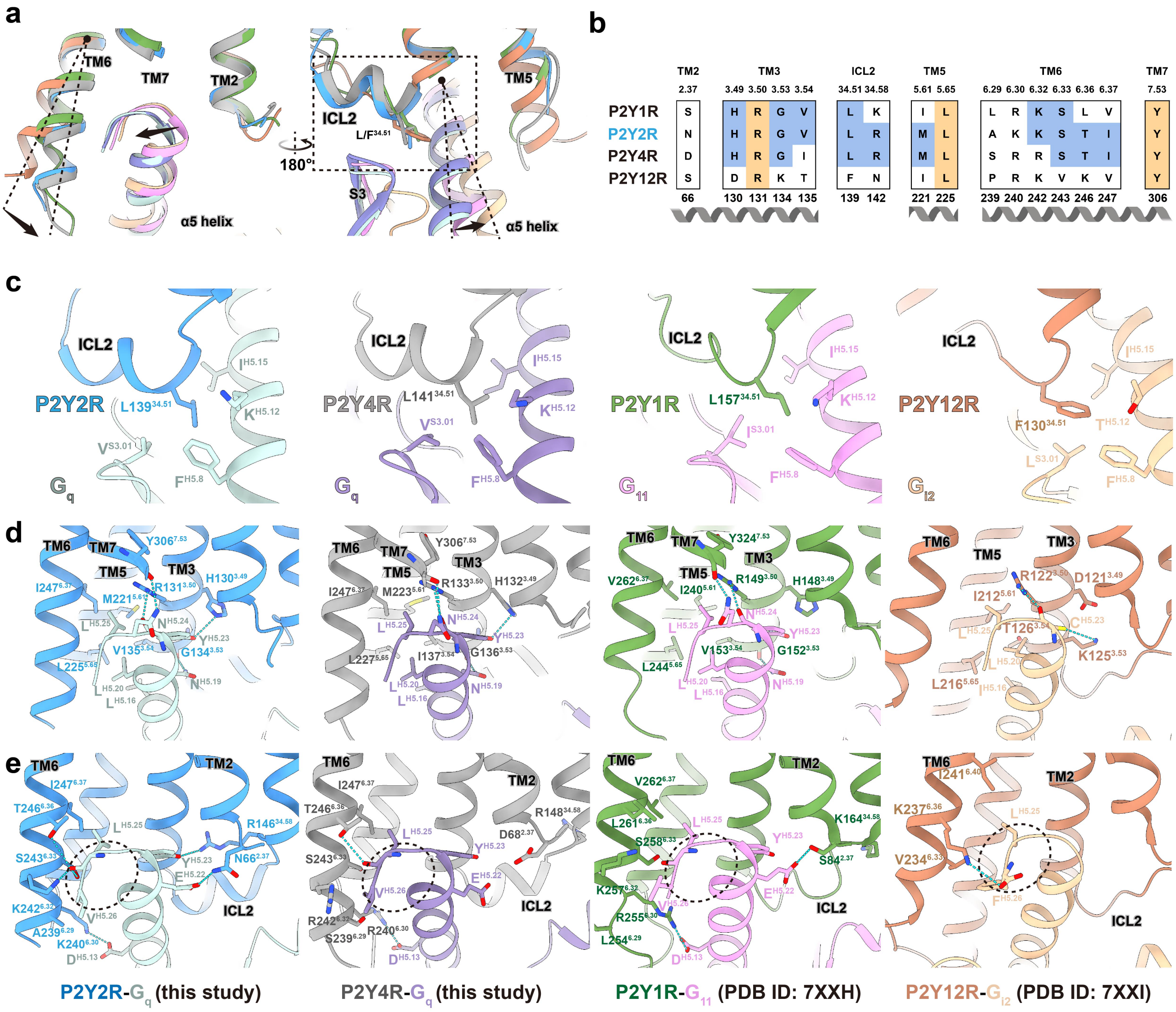

Supplementary Fig. S3 Comparison of the G-protein binding pockets of P2Y1R, P2Y2R, P2Y4R, and P2Y12R. a Structural overlay of P2Y2R-G_q, P2Y4R-G_q, P2Y1R-G_{11} (PDB ID: 7XXH) and P2Y12R-G_{i2} (PDB ID: 7XXI) complexes. Receptors are aligned. The black arrows indicate the movement of receptor TM6 and the rotational shift of the α5 helix of G-protein from the P2Y12R-G_{i2} complex to the P2Y2R-G_q complex. A representative residue L/$F^{34.51}$, which contributes to the displacement of α5 helix, is shown as sticks. Enlarged area in the panel (**c**) is depicted by a dashed rectangle. **b** Sequence alignment of the residues in the interface of P2Y1R-G_{11}, P2Y2R-G_q, P2Y4R-G_q, and P2Y12R-G_{i2} according to their cryo-EM structures. The conserved residues compared with P2Y2R are highlighted in light blue, and the conserved residues across all four P2Y receptors are highlighted in orange. The Ballesteros-Weinstein numbering for all P2Y receptors is labeled above the sequence, while the residue numbers for P2Y2R are indicated below the sequence. **c** Close-up view of the

interactions between L/F$^{34.51}$ of ICL2 and G-protein of P2Y2R-G_q, P2Y4R-G_q, P2RY1R-G_{11} and P2Y12R-G_{i2} complexes (from left to right). **d, e** Comparison of the detailed receptor-G-protein interactions in P2Y1R-G_{11}, P2Y2R-G_q, P2Y4R-G_q, and P2Y12R-G_{i2} complexes. The conserved (**d**) and specific (**e**) interactions in $G_{q/11}$ coupling of P2Y1R-like receptors are displayed. The corresponding residues in the P2Y12R-G_{i2} complex are shown in panel (**d**) and (**e**) for comparison. In panel **e**, the 'wavy hook' is highlighted by an elliptical dashed box.

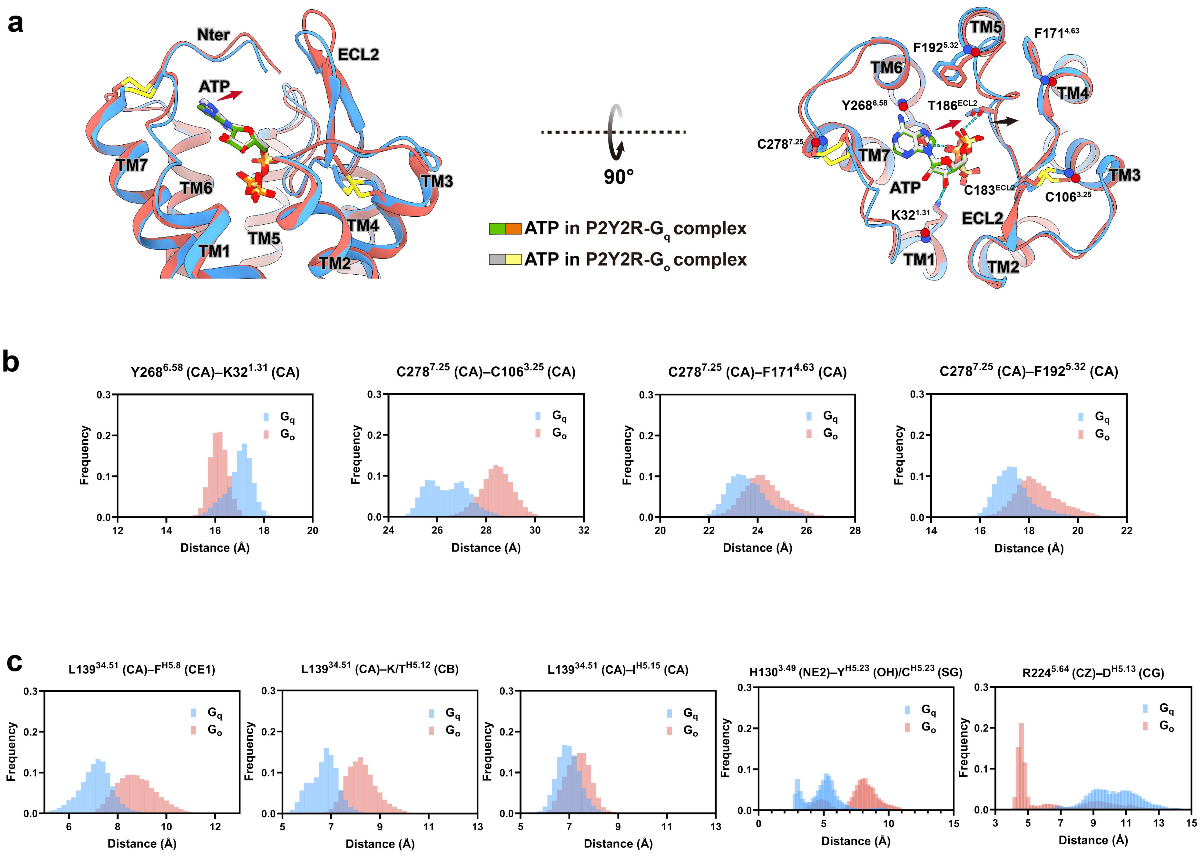

Supplementary Fig. S4 Conformational changes and interactions in ATP-P2Y2R-G_q and ATP-P2Y2R-G_o complexes revealed by MD simulations. a Comparison of the ATP binding pocket in the P2Y2R-G_q and P2Y2R-G_o complexes, viewed from the side and top, by superimposing the receptor structures. Key residues used for distance measurements (**b**) are depicted as sticks, with Cα atoms marked by blue (G_q) or red (G_o) dots. The shifts of ATP are indicated by red arrows. The displacements of ligand binding pocket in receptor are indicated by black arrows. Polar contacts are depicted as light blue dashed lines. **b** Distances between transmembrane (TM) helices in the ligand binding pocket of P2Y2R when coupled to G_q or G_o were quantified using molecular dynamics (MD) simulations. The frequency distribution histograms are used to illustrate the distance distributions for each coupled residue in a 3μs MD simulations. **c** Quantification of the key interactions between P2Y2R and G_q or G_o protein by distance distributions during a 3 μs MD simulations. The distance distributions are represented as frequency distribution histograms.

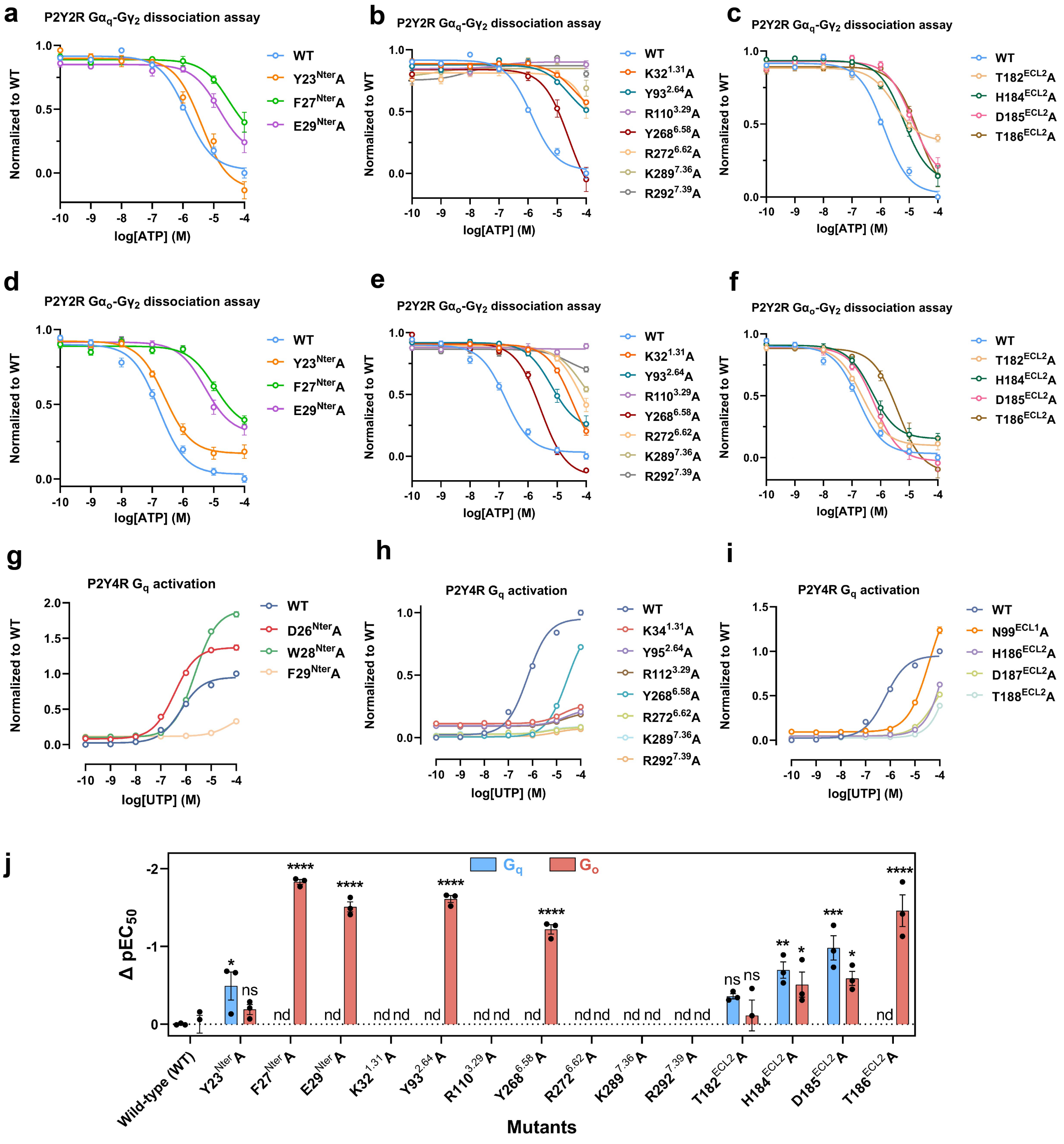

Supplementary Fig. S5 Contributions of ligand-binding pocket residues to G_q or G_o signaling in P2Y2R and P2Y4R. a-f The mutational effects of pocket residues on ATP-induced G_q (**a-c**) or G_o (**d-f**) coupling activity of P2Y2R were assessed using $G\alpha_q$-$G\gamma_2$ or $G\alpha_o$-$G\gamma_2$ dissociation assays, respectively. Each data point represents the mean ± s.e.m. from three independent experiments (n=3), with each experiment performed in triplicate. The dose-response curves illustrate the variations in potency (EC_{50}) and efficacy (E_{max}) between the wild-type (WT) P2Y2R and its alanine mutants. **g-i** The mutational effects of pocket residues on UTP-induced G_q coupling

activity in P2Y4R was assessed through a $G_{s/q}$-based cAMP accumulation assay. The dose-response curves illustrate the variations in potency (EC_{50}) and efficacy (E_{max}) when comparing the WT P2Y4R to its alanine mutants. **j** A column chart illustrates the potency changes of ligand binding pocket residue mutants of the P2Y2R relative to its wild-type form when coupling to G_q or G_o proteins under the treatment of ATP. The ΔpEC50 values represent the difference in pEC50 between each mutant and the wild-type P2Y2R. Statistical differences in ΔpEC50 values were assessed using one-way ANOVA followed by Dunnett's multiple comparisons test, comparing each mutant to the wild-type (WT) P2Y2R. "nd" indicates that the value was not determined. "ns" denotes no significant difference. Significance levels are indicated as follows: **** $p < 0.0001$, *** $p < 0.001$, ** $p < 0.01$, and * $p < 0.05$. A comprehensive statistical analysis of mutation assay and expression levels of each mutant in P2Y2R and P2Y4R are provided in Supplementary Table S5.

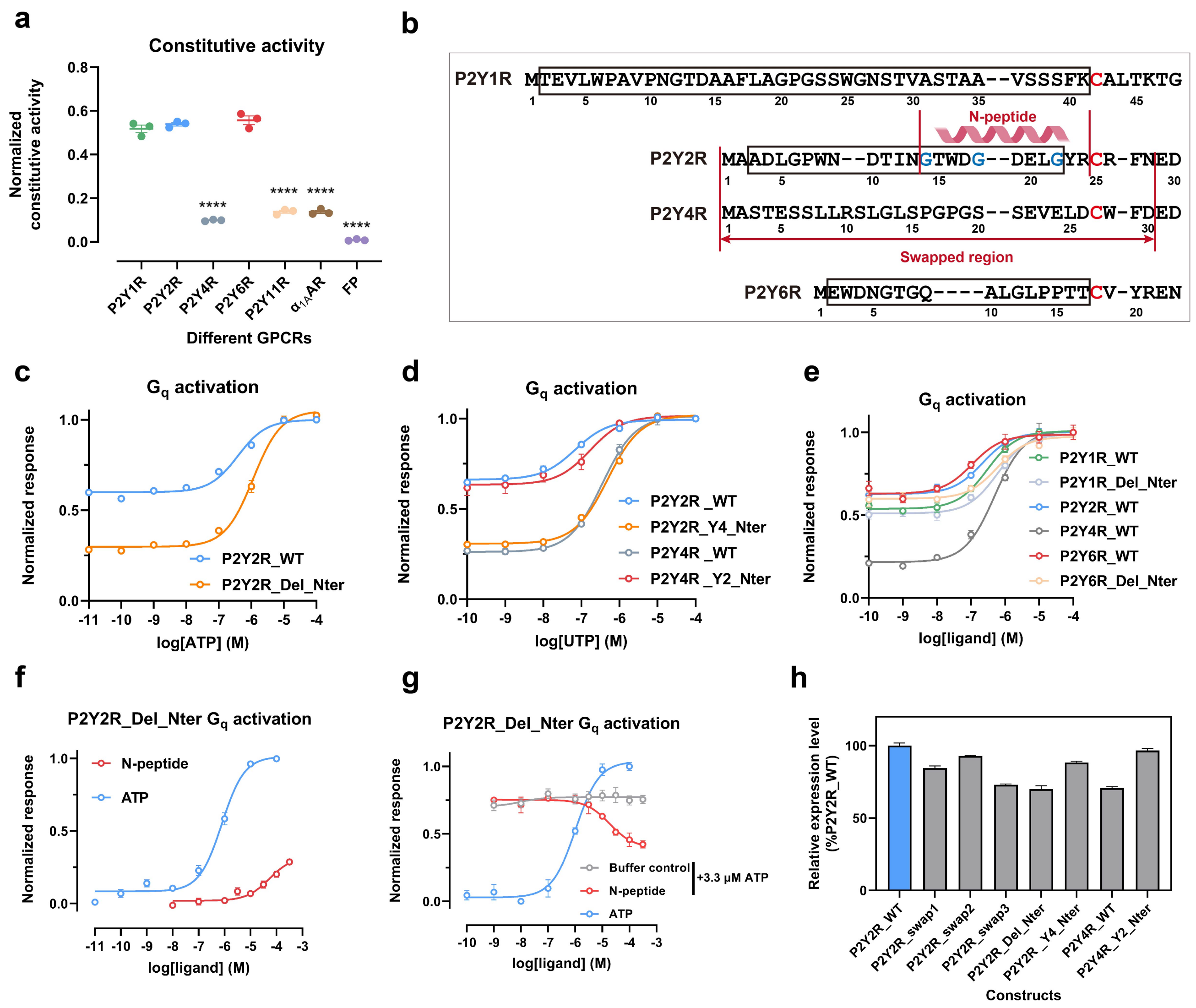

Supplementary Fig. S6 N-terminus contributes to the high constitutive activity of P2Y2R. a Comparison of constitutive activity of P2Y2R with G_q-coupled P2Y1R-like receptors and two other class A GPCRs: α1A-adrenergic receptor (α_{1A}AR) and prostaglandin F2α receptor (FP receptor). The constitutive activity of each receptor was determined using a $G_{s/q}$-based cAMP accumulation assay. It was normalized by the relative luminescence units (RFUs) of the vehicle treatment group to the luminescence value obtained from the control group (no receptor) and the full agonist-treated group. Each data represents the mean ± s.e.m. from three independent experimental replicates (n = 3), each performed in triplicate. **b** Sequence alignment of the N-terminus for P2Y1R, P2Y2R, P2Y4R, and P2Y6R is shown, with emphasis on the N-peptide segment of P2Y2R and engineered regions. The truncated regions in P2Y1R, P2Y2R, and P2Y6R are highlighted by black rectangles, and the swapped region between P2Y2R and P2Y4R is marked by red separation lines. **c** Comparison

of the constitutive activity between P2Y2R N-terminus truncated mutant (P2Y2R_Del_Nter) and wild-type form (P2Y2R_WT). **d** Effects of N-terminus mutual-swaps between P2Y2R and P2Y4R on their constitutive activity. The effects are evaluated by comparing the N-terminus swapped mutants (P2Y2R_Y4Nter and P2Y4R_Y2Nter) with their respective wild-type forms (P2Y2R_WT and P2Y4R_WT). **e** Effects of N-terminus truncation on the constitutive activity of P2Y1R and P2Y6R. The effects are evaluated by comparing the N-terminus truncated mutants (P2Y1R_Del_Nter and P2Y6R_Del_Nter) with their respective wild-type forms (P2Y1R_WT and P2Y6R_WT), using P2Y2R_WT and P2Y4R_WT as controls. For panel **c-e**, the dose-response curves of normalized RLUs under treatment with varying concentrations of agonist are presented for each receptor to reflect their constitutive activity. Each data represents the mean ± s.e.m. from three independent experimental replicates (n = 3). **f** Dose response curves of the P2Y2R N-terminus truncated mutant (P2Y2R_Del_Nter) under treatment with a synthesized N-peptide and ATP. Each data represents the mean ± s.e.m. from three independent replicates (n = 3), each performed in duplicate. **g** Antagonistic effect of the N-peptide on P2Y2R_Del_Nter G_q activation under treatment with 3.3 μM ATP. Each data represents the mean ± s.e.m. from three independent replicates (n = 3). **h** Relative expression levels of N-terminus truncated or swapped mutants of P2Y2R and P2Y4R, compared with their wild-type forms. Each data represents the mean ± s.e.m. from three independent replicates (n = 3). A detailed statistical evaluation for above assays is listed in Supplementary Tables S9-S12.

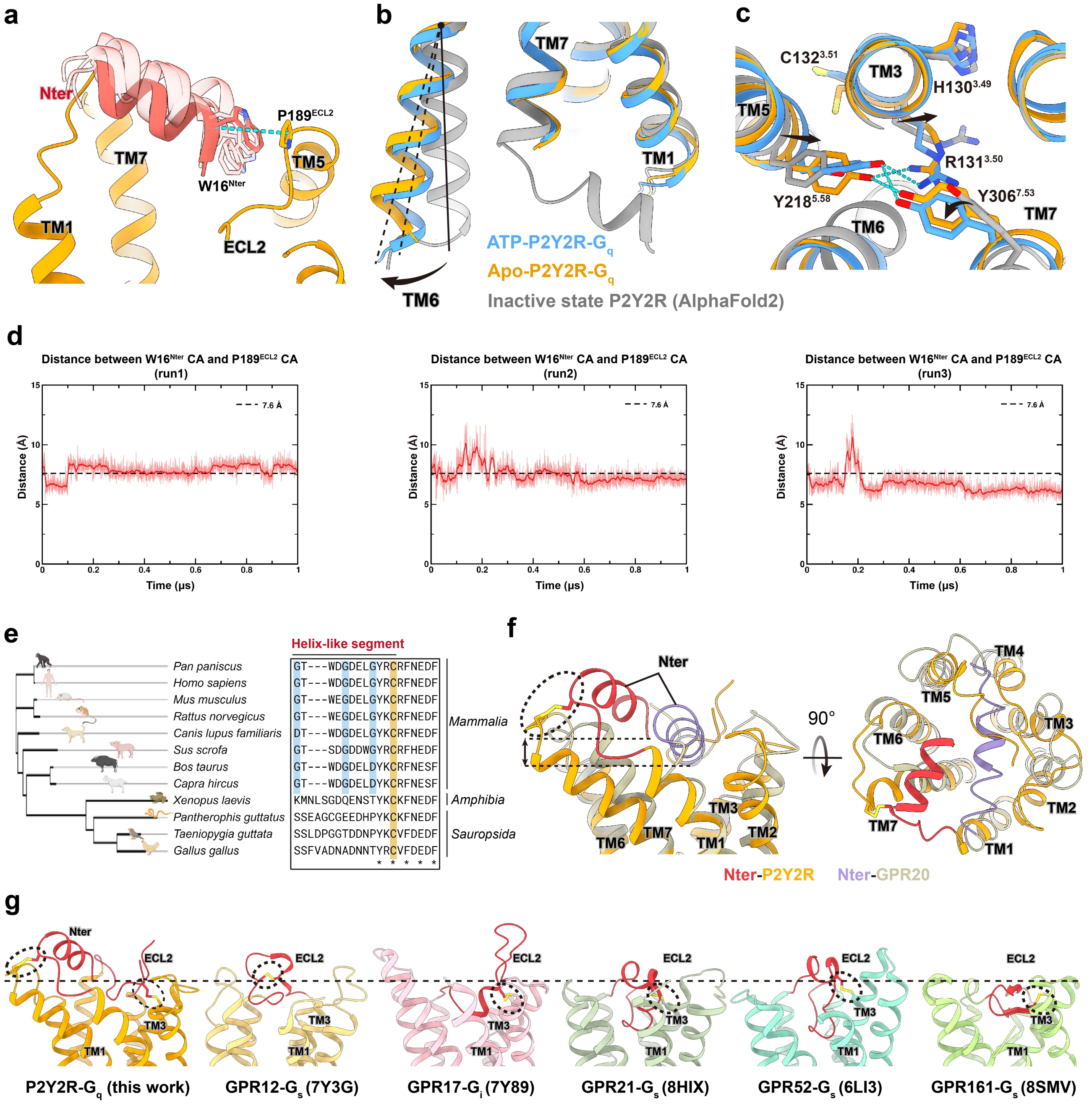

Supplementary Fig. S7 Characterization of N-terminus-induced self-activation of P2Y2R using MD simulations and structural comparisons. a Molecular dynamics simulations revealed the interactions between N-terminus and ligand-binding pocket in P2Y2R. The N-terminus is highlighted in light red in three representative structures obtained from three independent MD simulation runs. For comparison, the cryo-EM structure is shown in dark red and orange. A representative interaction between residues W16^{Nter} and P189$^{\mathrm{ECL2}}$ is depicted as sticks. **b** Structural superposition of the P2Y2R structures in the ATP-induced full active state, the N-terminus-induced self-activation state, and the AlphaFold2-predicted inactive state. The activation-relevant

outward displacement of TM6 is indicated by a black arrow. **c** A close-up view showing the close packing of R131$^{3.50}$, Y218$^{5.58}$, and Y306$^{7.53}$ of P2Y2R in both active states. Conformational changes in TM3, TM5, and TM7 are indicated by black arrows, and hydrogen bonds or salt bridges are depicted as blue dashed lines. **d** Time trace plot of the distances between the residues W16^{Nter} and P189^{ECL2} in three independent MD simulation runs, each lasting 1 μs. A dashed black horizontal line drawn at 7.6 Å indicates the distance observed in the cryo-EM structure, providing a reference for comparison with the dynamic features of the residues during the simulations. **e** Sequence alignment of the helix-like region in P2Y2R N-terminus across different vertebrate species, highlighting the evolutionary conservation of this region in *Mammalia* from the evolutionary tree. **f** Comparison of the N-terminus occupation of P2Y2R with the self-activating GPR20, viewed from the side (left) and top (right). A dashed horizontal line dashed drawn from the bottom of P2Y2R N-terminus binding pocket is serves as a reference to compare the binding pose of N-terminus in both receptors. Disulfide bonds that constrain the conformation of the N-terminus or ECL2 are depicted as yellow sticks and highlighted within a black dashed circle. **g** Comparison of the binding pose of N-terminus and ECL2 in P2Y2R with ECL2 in several self-activating orphan GPCRs. The selected orphan GPCRs, encompassing GPR12, GPR17, GPR21, GPR52, and GPR161 (from left to right), are reported to exhibit constitutive activity induced by ECL2. The N-terminus and ECL2 are colored in red. Disulfide bonds that constrain the conformation of the N-terminus or ECL2 are depicted as yellow sticks and highlighted within a black dashed circle.

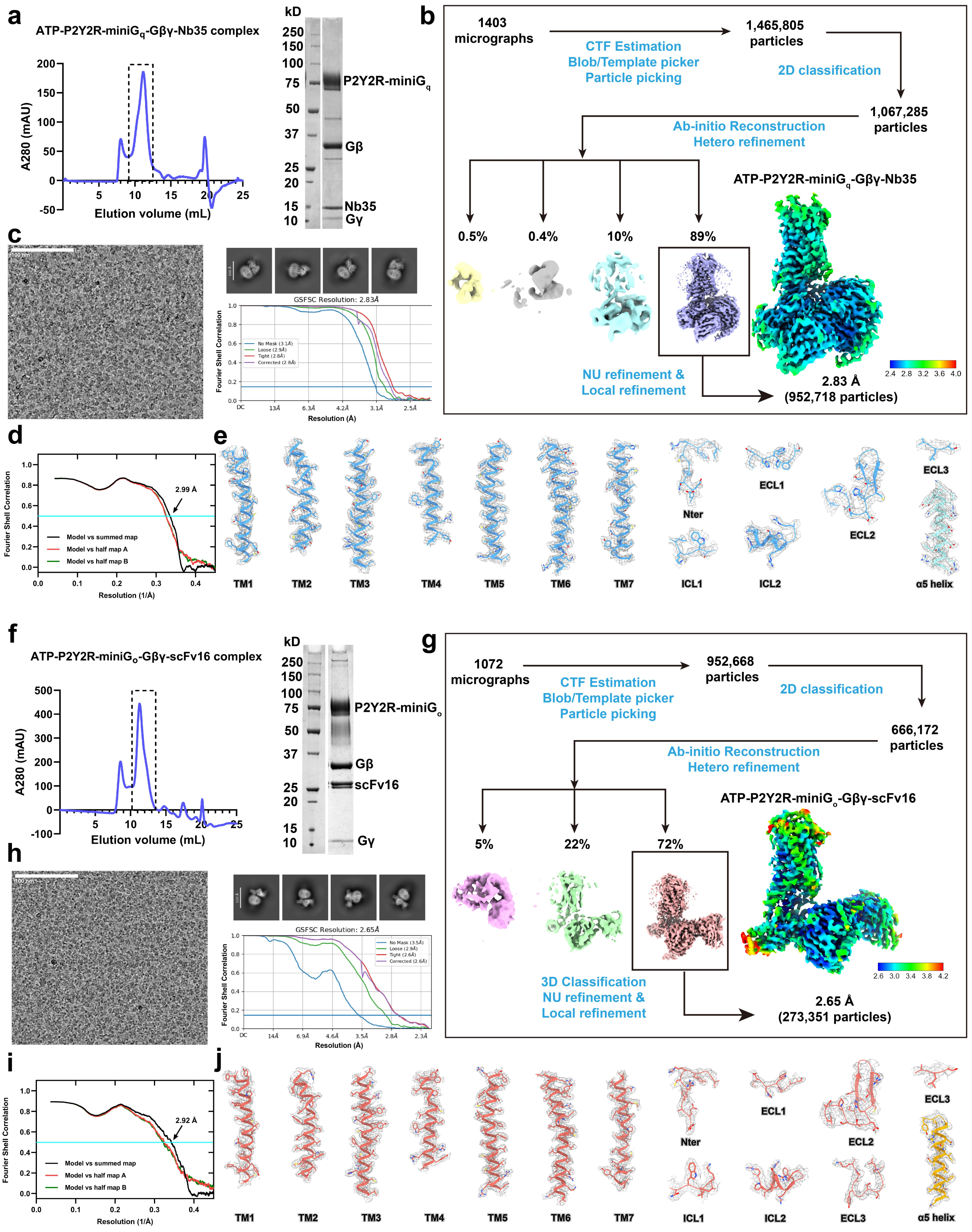
a
ATP-P2Y2R-miniGq-Gβγ-Nb35 complex
A280 (mAU)
Elution volume (mL)
kD
P2Y2R-miniGq
Gβ
Nb35
Gγ
b
1403 micrographs
CTF Estimation
Blob/Template picker
Particle picking
1,465,805 particles
2D classification
1,067,285 particles
Ab-initio Reconstruction
Hetero refinement
0.5%
0.4%
10%
89%
ATP-P2Y2R-miniGq-Gβγ-Nb35
NU refinement &
Local refinement
2.83 Å
(952,718 particles)
c
GSFSC Resolution: 2.83Å
Fourier Shell Correlation
Resolution (Å)
d
2.99 Å
Model vs summed map
Model vs half map A
Model vs half map B
Resolution (1/Å)
e
TM1
TM2
TM3
TM4
TM5
TM6
TM7
Nter
ICL1
ECL1
ICL2
ECL2
ECL3
α5 helix
f
ATP-P2Y2R-miniGo-Gβγ-scFv16 complex
A280 (mAU)
Elution volume (mL)
kD
P2Y2R-miniGo
Gβ
scFv16
Gγ
g
1072 micrographs
CTF Estimation
Blob/Template picker
Particle picking
952,668 particles
2D classification
666,172 particles
Ab-initio Reconstruction
Hetero refinement
5%
22%
72%
ATP-P2Y2R-miniGo-Gβγ-scFv16
3D Classification
NU refinement &
Local refinement
2.65 Å
(273,351 particles)
h
GSFSC Resolution: 2.65Å
Fourier Shell Correlation
Resolution (Å)
i
2.92 Å
Model vs summed map
Model vs half map A
Model vs half map B
Resolution (1/Å)
j
TM1
TM2
TM3
TM4
TM5
TM6
TM7
Nter
ICL1
ECL1
ICL2
ECL2
ECL3
α5 helix

Supplementary Fig. S8 Biochemistry and Cryo-EM data processing of ATP-P2Y2R-miniG$_q$-Nb35 and ATP-P2Y2R-miniG$_o$-scFv16 complexes. a, f Diagrams of size-exclusion chromatography and Coomassie blue stained SDS-PAGE gels of ATP-P2Y2R-miniG$_q$-Gβγ-Nb35 complex (**a**) and ATP-P2Y2R-miniG$_o$-scFv16 complex (**f**). The peak highlighted by a black dashed box was concentrated for making Cryo-EM samples. The raw images of the gels are provided in the Supplementary Fig. S10. **b, g** Workflow schematic of cryo-EM data processing for ATP-P2Y2R-miniG$_q$-Gβγ-Nb35 complex (**b**) and ATP-P2Y2R-miniG$_o$-Gβγ-scFv16 complex (**g**). All movies were motion-corrected by MotionCor2 and imported into cryoSPARC (v.3.1) for data processing. The final 3D density maps are colored according to local resolution (in Å) of the complexes. **c, h** Representative motion-corrected cryo-EM micrographs (left), two-dimensional (2D) class averages (top right), and Gold-standard Fourier Shell Correlation (GSFSC) curves (bottom right) of ATP-P2Y2R-miniG$_q$-Gβγ-Nb35 complex (**c**) and ATP-P2Y2R-miniG$_o$-Gβγ-scFv16 complex (**h**). **d, i** The Fourier Shell Correlation (FSC) curves for cross-validation between the final refined atomic model with the cryo-EM density maps. Specifically, the FSC curves between the same model and the map reconstructed from all particles (black), the map reconstructed from the half particles (red), and the map reconstructed from the other half particles (green) are shown for ATP-P2Y2R-miniG$_q$-Gβγ-Nb35 complex (**d**) and ATP-P2Y2R-miniG$_o$-Gβγ-scFv16 complex (**i**). **e, j** Local cryo-EM density for the final 3D refined maps of ATP-P2Y2R-miniG$_q$-Gβγ-Nb35 structure (**e**) and ATP-P2Y2R-miniG$_o$-Gβγ-scFv16 structure (**j**). The density maps and models of the transmembrane (TM) helices, N-terminus, extracellular loops, and intracellular loops from receptor and α5 helix from miniG$_q$ or miniG$_o$ proteins are shown using the global maps. The density is depicted as gray mesh using the Chimera X software.

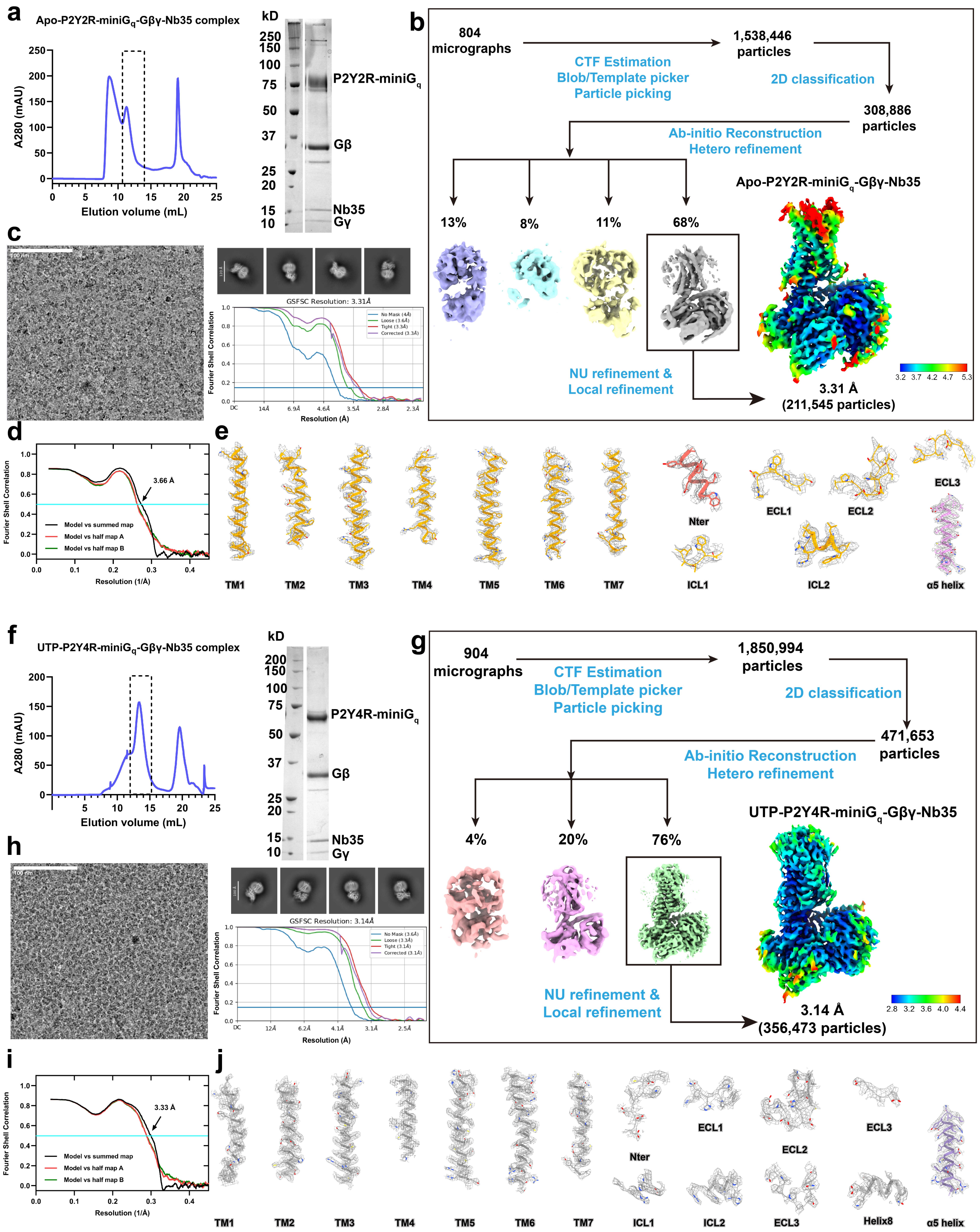
a
Apo-P2Y2R-miniGq-Gβγ-Nb35 complex
A280 (mAU)
Elution volume (mL)
kD
P2Y2R-miniGq
Gβ
Nb35
Gγ
b
804 micrographs
1,538,446 particles
CTF Estimation
Blob/Template picker
Particle picking
2D classification
308,886 particles
Ab-initio Reconstruction
Hetero refinement
13%
8%
11%
68%
Apo-P2Y2R-miniGq-Gβγ-Nb35
NU refinement &
Local refinement
3.31 Å
(211,545 particles)
c
GSFSC Resolution: 3.31Å
Fourier Shell Correlation
Resolution (Å)
d
3.66 Å
Model vs summed map
Model vs half map A
Model vs half map B
Resolution (1/Å)
e
TM1
TM2
TM3
TM4
TM5
TM6
TM7
Nter
ICL1
ECL1
ECL2
ICL2
ECL3
α5 helix
f
UTP-P2Y4R-miniGq-Gβγ-Nb35 complex
A280 (mAU)
Elution volume (mL)
kD
P2Y4R-miniGq
Gβ
Nb35
Gγ
g
904 micrographs
1,850,994 particles
CTF Estimation
Blob/Template picker
Particle picking
2D classification
471,653 particles
Ab-initio Reconstruction
Hetero refinement
4%
20%
76%
UTP-P2Y4R-miniGq-Gβγ-Nb35
NU refinement &
Local refinement
3.14 Å
(356,473 particles)
h
GSFSC Resolution: 3.14Å
Fourier Shell Correlation
Resolution (Å)
i
3.33 Å
Model vs summed map
Model vs half map A
Model vs half map B
Resolution (1/Å)
j
TM1
TM2
TM3
TM4
TM5
TM6
TM7
Nter
ICL1
ECL1
ICL2
ECL2
ECL3
ECL3
Helix8
α5 helix

Supplementary Fig. S9 Biochemistry and Cryo-EM data processing of apo-P2Y2R-miniG$_q$-Nb35 and UTP-P2Y4R-miniG$_q$-Nb35 complexes. a, f Diagrams of size-exclusion chromatography and Coomassie blue stained SDS-PAGE gels of apo-P2Y2R-miniG$_q$-Nb35 complex (**a**) and UTP-P2Y4R-miniG$_q$-Nb35 complex (**f**). The peak highlighted by a black dashed box was concentrated for making Cryo-EM samples. The raw images of the gels are provided in the Supplementary Fig. S10. **b, g** Workflow schematic of cryo-EM data processing for apo-P2Y2R-miniG$_q$-Nb35 complex (**b**) and UTP-P2Y4R-miniG$_q$-Nb35 complex (**g**). All movies were motion-corrected by MotionCor2 and imported into cryoSPARC (v.3.1) for data processing. The final 3D density maps are colored according to local resolution (in Å) of the complexes. **c, h** Representative motion-corrected cryo-EM micrographs (left), two-dimensional (2D) class averages (top right), and Gold-standard Fourier Shell Correlation (GSFSC) curves (bottom right) of apo-P2Y2R-miniG$_q$-Nb35 complex (**c**) and UTP-P2Y4R-miniG$_q$-Nb35 complex (**h**). **d, i** The Fourier Shell Correlation (FSC) curves for cross-validation between the final refined atomic model with the cryo-EM density maps. Specifically, the FSC curves between the same model and the map reconstructed from all particles (black), the map reconstructed from the half particles (red), and the map reconstructed from the other half particles (green) are shown for apo-P2Y2R-miniG$_q$-Nb35 complex (**d**) and UTP-P2Y4R-miniG$_q$-Nb35 complex (**i**). **e, j** Local cryo-EM density for the final 3D refined maps of apo-P2Y2R-miniG$_q$-Nb35 structure (**e**) and UTP-P2Y4R-miniG$_q$-Nb35 structure (**j**). The density maps and models of the transmembrane (TM) helices, N-terminus, extracellular loops, and intracellular loops from receptor and α5 helix from miniG$_q$ are shown using the global maps. The density is depicted as gray mesh using the Chimera X software.

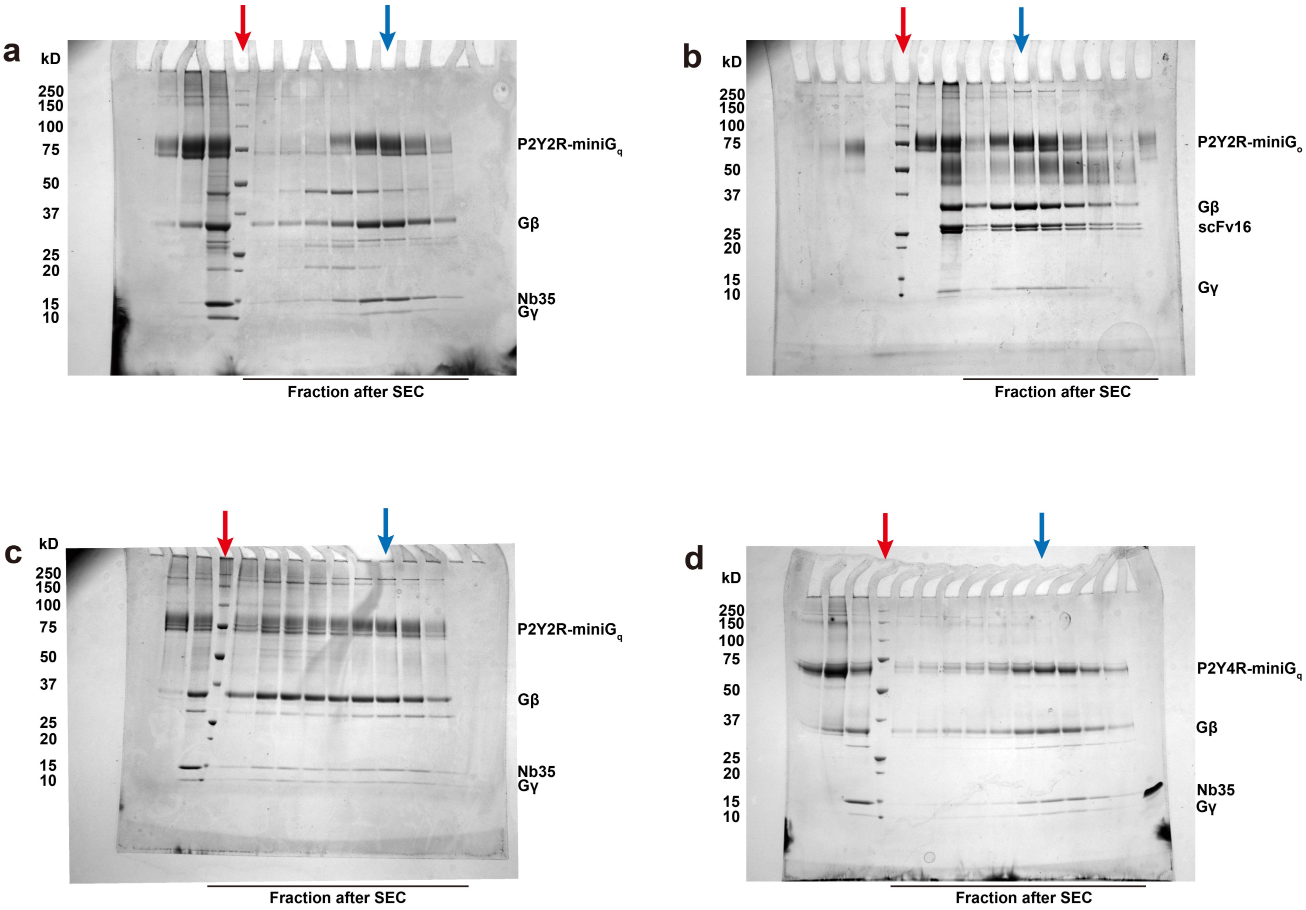

Supplementary Fig. S10 Uncropped SDS-PAGE gels. Coomassie-stained gels of ATP-P2Y2R-miniG$_q$-Gβγ-Nb35 (**a**), ATP-P2Y2R-miniG$_o$-Gβγ-scFv16 (**b**), Apo-P2Y2R-miniG$_q$-Gβγ-Nb35 (**c**), and UTP-P2Y4R-miniG$_q$-Gβγ-Nb35 (**d**) complexes. Red arrows indicate the marker lanes, and blue arrows point to the lanes of the target complexes shown in Supplementary Figs. S8 and S9.

Supplementary Tables S1-S16

Supplementary Table S1. Signaling profiles of P2Y1R, P2Y2R, and P2Y4R in response to different ligands.

Constructs	Assay	Ligands	G-protein subtypes	pEC_{50}	E_{max} (%)	n
Wild-type P2Y2R	NanoBit-based G-protein dissociation	ATP	G_q	5.83 ± 0.03	100.0 ± 1.2	3
		ADP	G_q	4.82 ± 0.02 ****	71.5 ±0.9 ***	3
		UTP	G_q	5.89 ± 0.02	101.4 ± 0.7	3
Wild-type P2Y1R	$G_{s/q}$-based cAMP accumulation	ATP	G_q	6.01 ± 0.05 ***	92.5 ± 1.1 *	3
		ADP	G_q	6.78 ± 0.03	102.9 ± 2.1	3
		UTP	G_q	N.D.	N.D.	3
Wild-type P2Y4R	$G_{s/q}$-based cAMP accumulation	ATP	G_q	4.93 ± 0.01 ****	79.9 ± 1.4 ****	3
		ADP	G_q	5.38 ± 0.21 ****	71.2 ± 0.4 ****	3
		UTP	G_q	6.30 ± 0.02	97.7 ± 0.3	3

The data are presented as mean pEC_{50} ± s.e.m. or mean E_{max} ± s.e.m. from three independent biological replicates. For each receptor, mean pEC_{50} and E_{max} of different ligands-induced receptor activation are compared. Statistical differences of each treated group from ATP-induced P2Y2R or UTP-induced P2Y4R activation are performed using one-way ANOVA followed by Dunnett's multiple comparison test. Statistical differences of each treated group from ADP-treated P2Y1R are determined using Student's t-test. "N.D." indicates that the value is not determined. Significance levels are denoted as follows: ****$p < 0.0001$, ***$p < 0.001$, **$p < 0.01$, *$p < 0.05$.

Supplementary Table S2. G-protein subtype coupling studies of P2Y2R through G-protein dissociation assay.

Constructs	Assay	Ligands	G-protein subtypes	pEC_{50}	E_{max} (%)	n
Wild-type P2Y2R	NanoBit-based G-protein dissociation	ATP	G_s	N.D.	N.D.	3
			G_q	5.81 ± 0.03	76.0 ± 0.4	3
			G_{12}	6.06 ± 0.02 *	74.3 ± 0.8	3
			G_{13}	5.90 ± 0.01	99.7 ± 0.6 ****	3
			G_{i1}	7.00 ± 0.08 ****	38.5 ±1.0 ****	3
			G_{i2}	6.67 ± 0.09 ****	34.0 ± 3.9 ****	3
			G_{i3}	N.D.	N.D.	3
			G_o	6.75 ± 0.04 ****	70.3 ± 1.1	3

Data are represented as mean pEC_{50} ± s.e.m. or mean E_{max} ± s.e.m. from three independent biological replicates. Statistical differences of mean pEC_{50} and E_{max} values are assessed using one-way ANOVA followed by Dunnett's multiple comparisons test, comparing the coupling of each G-protein subtype to G_q protein. "N.D." indicates that the value is not determined. Significance levels are denoted as follows: **** $p < 0.0001$, *** $p < 0.001$, ** $p < 0.01$, * $p < 0.05$.

Supplementary Table S3. Summary of mutagenesis studies of activation-related residues in P2Y2R.

Ligands	Assay	G-protein subtypes	Constructs	pEC_{50}	E_{max} (%WT)	n	Expression level, n=3 (%WT)
ATP	NanoBit-based G-protein dissociation	G_q	WT	6.15 ± 0.06	100.0 ± 1.1	3	100.0 ± 2.8
			$C25^{Nter}A$	N.D.	N.D.	3	48.5 ± 1.2
			$F261^{6.51}A$	5.50 ± 0.05 ****	120.4 ± 1.6 ****	3	78.7 ± 1.2
			$N285^{7.32}A$	5.49 ± 0.03 ****	108.6 ± 0.6 *	3	101.1 ± 0.5
			$Y288^{7.35}A$	N.D.	N.D.	3	90.4 ± 1.1
			$Y306^{7.53}A$	5.04 ± 0.03 ****	83.1 ± 2.7 ***	3	81.1 ± 0.8

The mutational effects of key residues in the N-terminus, TM6, and TM7 of P2Y2R on G_q activation were investigated through the G-protein dissociation assay. Data are represented as mean pEC_{50} ± s.e.m. or mean E_{max} ± s.e.m. from three independent biological replicates. Statistical differences of mean pEC_{50} or E_{max} values were assessed using one-way ANOVA followed by Dunnett's multiple comparisons test, comparing each mutant to the wild-type (WT) P2Y2R in response to ATP. "N.D." indicates that the value was not determined. Significance levels are denoted as follows: **** $p < 0.0001$, *** $p < 0.001$, ** $p < 0.01$, * $p < 0.05$. The expression levels of each mutant were tested by a HiBiT-based assay and were shown as a proportion of the expression level of wild-type P2Y2R.

Supplementary Table S4. Summary of the mutagenesis studies in the N-terminus of P2Y2R and P2Y4R.

Assay	Constructs	G-protein subtypes	Ligands	pEC_{50}	n	Expression level, n=3 (%WT)
$G_{s/q}$-based cAMP accumulation	P2Y2R_WT	G_q	ATP	6.80 ± 0.04	3	100.0 ± 1.9
	P2Y2R_Nter3M	G_q	ATP	5.80 ± 0.02 ****	3	89.1 ± 1.2
	P2Y4R_WT	G_q	ATP	4.99 ± 0.02	3	77.8 ± 0.9
	P2Y4R_Nter3M	G_q	ATP	5.50 ± 0.03 ****	3	84.2 ± 0.9

The mutagenesis studies of the P2Y2R and P2Y4R, as well as their N-terminal reciprocal mutants P2Y2R_Nter3M and P2Y4R_Nter3M, were conducted to elucidate the functional significance of the N-terminus in ATP recognition. The data are presented as the mean pEC_{50} ± s.e.m. from three independent biological replicates. Statistical differences in mean pEC_{50} values are evaluated using one-way ANOVA followed by Dunnett's multiple comparisons test, comparing each mutant to the respective wild-type (WT) P2Y2R or P2Y4R in response to ATP. Significance levels are denoted as follows: **** $p < 0.0001$, *** $p < 0.001$, ** $p < 0.01$, * $p < 0.05$. The expression levels of the mutants were quantified using a HiBiT-based assay and were reported as a percentage of the expression level of wild-type P2Y2R.

Supplementary Table S5. Summary of mutagenesis studies of pocket residues in P2Y2R and P2Y4R.

Receptor	Ligands	Assay	Constructs	G-protein subtypes	pEC$_{50}$	E$_{max}$ (%WT)	n	Expression level, n=3 (%WT)
P2Y2R	ATP	NanoBit-based G-protein dissociation	Wild-type (WT)	G_q	5.88 ± 0.01	100.0 ± 7.2	9	100.0 ± 2.8
				G_o	6.78 ± 0.11	100.0 ± 2.1	9	
			$Y23^{Nter}A$	G_q	5.39 ± 0.18 *	116.0 ± 11.1	9	83.9 ± 3.3
				G_o	6.59 ± 0.06	85.6 ± 8.7	9	
			$F27^{Nter}A$	G_q	N.D.	N.D.	9	118.9 ± 4.6
				G_o	4.95 ± 0.03 ****	68.3 ± 2.3	9	
			$E29^{Nter}A$	G_q	N.D.	N.D.	9	80.4 ± 2.0
				G_o	5.27 ± 0.06 ****	71.9 ± 10.8	9	
			$K32^{1.31}A$	G_q	N.D.	N.D.	9	96.4 ± 0.5
				G_o	N.D.	N.D.	9	
			$Y93^{2.64}A$	G_q	N.D.	N.D.	9	72.8 ± 0.5
				G_o	5.17 ± 0.05 ****	80.9 ± 14.1	9	
			$R110^{3.29}A$	G_q	N.D.	N.D.	9	104.3 ± 1.3
				G_o	N.D.	N.D.	9	
			$Y268^{6.58}A$	G_q	N.D.	N.D.	9	101.5 ± 3.9
				G_o	5.56 ± 0.06 ****	119.9 ± 3.3	9	
			$R272^{6.62}A$	G_q	N.D.	N.D.	9	106.2 ± 1.7
				G_o	N.D.	N.D.	9	
			$K289^{7.36}A$	G_q	N.D.	N.D.	9	102.7 ±2.2
				G_o	N.D.	N.D.	9	
			$R292^{7.39}A$	G_q	N.D.	N.D.	9	123.3 ± 3.3
				G_o	N.D.	N.D.	9	
			$T182^{ECL2}A$	G_q	5.52 ± 0.03	63.2 ± 4.9	9	83.0 ± 0.8
				G_o	6.67 ± 0.20	93.8 ± 10.6	9	
			$H184^{ECL2}A$	G_q	5.19 ± 0.11 **	91.8 ± 13.4	9	97.8 ± 3.1
				G_o	6.27 ± 0.16 *	88.0 ± 7.9	9	
			$D185^{ECL2}A$	G_q	4.90 ± 0.16 ***	91.6 ± 14.0	9	92.7 ± 1.1
				G_o	6.19 ± 0.09 *	106.3 ± 12.4	9	
			$T186^{ECL2}A$	G_q	N.D.	N.D.	9	110.2 ± 1.7
				G_o	5.32 ± 0.20 ****	119.1 ± 13.7	9	
P2Y4R	UTP	$G_{s/q}$-based cAMP accumulation	Wild-type (WT)	G_q	6.20 ± 0.01	100.0 ± 0.4	3	100.0 ± 0.4
			$D26^{Nter}A$	G_q	6.45 ± 0.03	144.0 ± 1.2 ****	3	118.4 ± 1.2
			$W28^{Nter}A$	G_q	5.64 ± 0.01 **	199.5 ± 1.7 ****	3	95.4 ± 0.7
			$F29^{Nter}A$	G_q	N.D.	N.D.	3	84.3 ± 0.8
			$K34^{1.31}A$	G_q	N.D.	N.D.	3	81.6 ± 0.4
			$Y95^{2.64}A$	G_q	N.D.	N.D.	3	83.5 ± 1.0
			$R112^{3.29}A$	G_q	N.D.	N.D.	3	98.3 ± 0.7
			$Y268^{6.58}A$	G_q	N.D.	N.D.	3	106.8 ± 0.8
			$R272^{6.62}A$	G_q	5.44 ± 0.21 ***	8.6 ± 0.2 ****	3	104.9 ± 0.4
			$K289^{7.36}A$	G_q	5.39 ± 0.09 ***	9.3 ± 0.3 ****	3	100.4 ± 0.8
			$R292^{7.39}A$	G_q	5.07 ± 0.08 ****	7.8 ± 0.3 ****	3	104.4 ± 1.2
			$N99^{ECL1}A$	G_q	N.D.	N.D.	3	99.3 ± 1.1
			$H186^{ECL2}A$	G_q	N.D.	N.D.	3	106.2 ± 1.1
			$D187^{ECL2}A$	G_q	N.D.	N.D.	3	95.2 ± 0.6
			$T188^{ECL2}A$	G_q	N.D.	N.D.	3	94.6 ± 0.5

Mutagenesis studies were conducted on the P2Y2R and P2Y4R to investigate the functional roles of key pocket residues involved in ligand recognition. For the P2Y2R, mutagenesis was studied through a G-protein

dissociation assay (G_q and G_o pathways). Data are represented as the mean pEC_{50} ± s.e.m. or mean E_{max} ± s.e.m. from three independent biological replicates, each performed in triplicate. For the P2Y4R, mutagenesis was studied through a $G_{s/q}$-based cAMP accumulation assay. Data are represented as the mean pEC_{50} ± s.e.m. or mean E_{max} ± s.e.m. from three independent biological replicates. Statistical differences in mean pEC_{50} or E_{max} values are assessed using one-way ANOVA followed by Dunnett's multiple comparisons test, comparing each mutant to the respective wild-type (WT) P2Y2R or P2Y4R in response to ATP or UTP, respectively. "N.D." indicates that the value was not determined. Significance levels are denoted as follows: **** $p < 0.0001$, *** $p < 0.001$, ** $p < 0.01$, * $p < 0.05$. The expression levels of each mutant were tested by a HiBiT-based assay and were shown as a proportion of the expression level of wild-type receptor.

Supplementary Table S6. Summary of mutagenesis studies of intracellular residues in P2Y2R associated with G-protein coupling.

Assay	Ligands	Constructs	G-protein subtypes	pEC_{50}	E_{max} (%WT)	n	Expression level, n=3 (%WT)
NanoBit-based G-protein dissociation	ATP	Wild-type (WT)	G_q	6.00 ± 0.04	100.0 ± 1.1	3	100.0 ± 0.7
			G_o	6.78 ± 0.01	100.0 ± 0.8	3	
		N66$^{2.37}$A	G_q	5.51 ± 0.03 ***	66.4 ± 0.4 ****	3	122.2 ± 1.9
			G_o	6.59 ± 0.03	90.4 ± 1.0 ***	3	
		H130$^{3.49}$A	G_q	5.38 ± 0.18 ****	27.6 ± 1.6 ****	3	110.2 ± 1.8
			G_o	6.77 ± 0.04	73.3 ± 1.2 ****	3	
		R131$^{3.50}$A	G_q	N.D.	N.D.	3	76.3 ± 0.5
			G_o	6.17 ± 0.09 ****	42.5 ± 0.6 ****	3	
		L139$^{34.51}$A	G_q	N.D.	N.D.	3	117.2 ± 1.7
			G_o	6.67 ± 0.04	67.6 ± 1.3 ****	3	
		R146$^{34.58}$A	G_q	5.81 ± 0.04	62.3 ±0.6 ****	3	112.2 ± 1.8
			G_o	6.84 ± 0.04	96.6 ± 1.2	3	
		R224$^{5.64}$A	G_q	5.68 ± 0.04 *	53.0 ± 2.9 ****	3	79.8 ± 2.1
			G_o	6.74 ±0.14	59.3 ± 1.7 ****	3	
		K240$^{6.30}$A	G_q	6.40 ± 0.03 **	53.5 ± 0.2 ****	3	93.3 ± 0.9
			G_o	6.55 ± 0.06	75.4 + 1.2 ****	3	
		K242$^{6.32}$A	G_q	5.91 ± 0.06	73.3 ± 0.9 ****	3	98.3 ± 1.5
			G_o	6.94 ± 0.03	86.3 ± 3.1 ****	3	
		S243$^{6.33}$A	G_q	5.44 ±0.04 ****	78.8 ± 1.2 ****	3	96.7 ± 1.0
			G_o	6.64 ± 0.07	92.8 ± 0.9 *	3	
		T246$^{6.36}$A	G_q	5.77 ± 0.04	45.8 ± 1.4 ****	3	110.5 ± 1.8
			G_o	6.88 ± 0.07	52.1 ± 0.7 ****	3	
		Y230/T232/S233^{ICL3}A	G_q	5.99 ± 0.03	87.1 ± 2.0 ****	3	105.9 ± 0.7
			G_o	6.23 ± 0.16 ***	46.2 ± 1.5 ****	3	

The effects of intracellular residues within the P2Y2R-G-protein interfaces on G_q and G_o activation were investigated using a G-protein dissociation assay. Data are presented as the mean pEC_{50} ± s.e.m. or mean E_{max}

± s.e.m. from three independent biological replicates. Statistical differences in mean pEC_{50} or E_{max} values are assessed using one-way ANOVA followed by Dunnett's multiple comparisons test, comparing each mutant to the wild-type (WT) P2Y2R in response to ATP. "N.D." indicates that the value was not determined. Significance levels are denoted as follows: **** $p < 0.0001$, *** $p < 0.001$, ** $p < 0.01$, * $p < 0.05$. The expression levels of each mutant were measured using a HiBiT assay and were reported as a percentage of the expression level of wild-type P2Y2R.

Supplementary Table S7. Summary of the constitutive activity studies of wild-type P2Y2R and its N-terminus swapped mutants.

Assay	Ligands	Constructs	Normalized constitutive activity (%)	n	Expression level, n=3 (%WT)
$G_{s/q}$-based cAMP accumulation	Full agonist: ATP<br>Inversed agonist: AR-C 118925XX	P2Y2R_WT	45.4 ± 0.6	3	100.0 ± 1.9
		P2Y2R_swap1	30.4 ± 0.1 ****	3	84.5 ± 1.6
		P2Y2R_swap2	23.9 ± 0.3 ****	3	92.8 ± 0.6
		P2Y2R_swap3	5.3 ± 0.1 ****	3	73.0 ± 0.6

The constitutive activity of wild-type (WT) P2Y2R and its N-terminus swapped mutants were evaluated using a $G_{s/q}$-based cAMP accumulation assay. The constitutive activity is normalized as described in the Methods section and is represented as the mean ± s.e.m. from three independent biological replicates. Statistical differences are assessed using one-way ANOVA followed by Dunnett's multiple comparisons test, comparing each mutant to the wild-type (WT) P2Y2R. Significance levels are denoted as follows: **** $p < 0.0001$, *** $p < 0.001$, ** $p < 0.01$, * $p < 0.05$. The expression levels of each mutant were quantified using a HiBiT assay and were expressed as a percentage of the WT P2Y2R's expression level.

Supplementary Table S8. Summary of the constitutive activity studies of wild-type P2Y2R and its single mutants.

Assay	Ligands	Constructs	Normalized constitutive activity (%WT)	n	Expression level, n=3 (%WT)
$G_{s/q}$-based cAMP accumulation	Full agonist: ATP Inversed agonist: AR-C 118925XX	Wild-type (WT)	100.0 ± 1.6	9	100.0 ± 2.8
		$C25^{Nter}A$	2.6 ± 0.5 ****	9	48.5 ± 1.2
		$H184^{ECL2}A$	20.1 ± 3.9 ****	9	97.8 ± 3.1
		$D185^{ECL2}A$	18.4 ± 3.7 ****	9	92.7 ± 1.1
		$P189^{ECL2}A$	81.2 ± 5.2 **	9	120.3 ± 0.6
		$F192^{5.32}A$	69.7 ± 4.9 ****	9	112.3 ± 0.4
		$Y268^{6.58}A$	42.5 ± 2.0 ****	9	101.5 ± 3.9
		$R272^{6.62}A$	22.6 ± 4.4 ****	9	106.2 ± 1.7
		$L276^{ECL3}A$	76.6 ± 2.4 ***	9	98.4 ± 4.3
		$C278^{7.25}A$	2.9 ± 0.5 ****	9	66.9 ± 1.7

The effects of alanine mutations of residues in the N-terminus, transmembrane helices (TMs), and ECL2 on the constitutive activity of P2Y2R were investigated using a $G_{s/q}$-based cAMP accumulation assay. The constitutive activity was calculated as described in the Methods section and is expressed as a percentage of the wild-type (WT) P2Y2R. Statistical differences of the mean constitutive activity are assessed using one-way ANOVA followed by Dunnett's multiple comparisons test, comparing each mutant to the wild-type (WT) P2Y2R. Significance levels are denoted as follows: **** $p < 0.0001$, *** $p < 0.001$, ** $p < 0.01$, * $p < 0.05$. The expression levels of each mutant were measured using a HiBiT assay and were reported as a percentage of the WT P2Y2R's expression level.

Supplementary Table S9. Summary of constitutive activity studies of different GPCRs.

Assay	Different GPCRs	Ligands	Normalized constitutive activity (%)	n	Expression level, n=3 (%WT)
$G_{s/q}$-based cAMP accumulation	P2Y1R	100 μM ADP	0.518 ± 0.017	9	172.5 ± 5.3
	P2Y2R	100 μM ATP	0.539 ± 0.008	9	100.0 ± 1.9
	P2Y4R	100 μM UTP	0.098 ± 0.002 ****	9	83.3 ± 3.3
	P2Y6R	100 μM UDP	0.557 ± 0.020	9	70.1± 2.9
	P2Y11R	100 μM ATP	0.138 ± 0.007 ****	9	114.9 ± 0.9
	$\alpha_{1A}AR$	10 μM Oxymetazoline	0.137 ± 0.007 ****	9	68.0 ± 1.8
	FP receptor	10 μM Latanoprost	0.008 ± 0.002 ****	9	48.1 ± 1.8

The constitutive activity of P2Y2R, along with other P2Y1R-like receptors and two additional Class A GPCRs, was evaluated using a $G_{s/q}$-based cAMP accumulation assay. The constitutive activity is normalized as described in the Methods section and is represented as the mean from three independent biological replicates, each

performed in triplicate. Statistical differences of mean constitutive activity are assessed using one-way ANOVA followed by Dunnett's multiple comparisons test, comparing each GPCR to the P2Y2R. Significance levels are denoted as follows: **** $p < 0.0001$, *** $p < 0.001$, ** $p < 0.01$, * $p < 0.05$. The expression levels of each receptor were measured using M1-FITC cell surface staining and were reported as a percentage of the WT P2Y2R's expression level.

Supplementary Table S10. Summary of the constitutive activity studies of wild-type P2Y2R, P2Y1R, P2Y6R, as well as their N-terminus truncated mutants.

Assay	Ligands	Receptor	Normalized constitutive activity (%)	n	Expression level, n=3 (%P2Y2R_WT)
$G_{s/q}$-based cAMP accumulation	ATP	P2Y2R_WT	60.0 ± 0.5	3	100.0 ± 1.9
		P2Y2R_Del_Nter	29.8 ± 0.3 ****	3	70.0 ± 2.3
	ADP	P2Y1R_WT	53.8 ± 0.5	3	130.0 ± 3.1
		P2Y1R_Del_Nter	51.1 ± 0.9	3	108.3 ± 3.3
	UDP	P2Y6R_WT	63.0 ± 0.8	3	67.9 ± 2.2
		P2Y6R_Del_Nter	59.9 ± 1.4	3	70.8 ± 3.1

The constitutive activity is normalized as the ratio of basal relative luminescence units (RFUs) to full activated RFUs, and corresponding values are represented as the mean from three independent biological replicates. Statistical differences in mean constitutive activity are assessed using one-way ANOVA followed by Tukey's multiple comparisons test, comparing each N-terminus truncated mutant to its corresponding wild-type form. Significance levels are denoted as follows: **** $p < 0.0001$, *** $p < 0.001$, ** $p < 0.01$, * $p < 0.05$. The expression level of each construct was measured using a HiBiT assay and was reported as a percentage of the wild-type P2Y2R 's expression level.

Supplementary Table 11. Summary of the constitutive activity studies of wild-type P2Y2R, P2Y4R, as well as their N-terminus swapped mutants.

Assay	Ligands	Receptor	Normalized constitutive activity (%)	n	Expression level, n=3 (%WT P2Y2R)
$G_{s/q}$-based cAMP accumulation	UTP	P2Y2R_WT	66.2 ± 0.4	3	100.0 ± 1.9
		P2Y2R_Y4_Nter	30.7 ± 0.2 ****	3	88.4 ± 0.9
		P2Y4R_WT	26.2 ± 0.1	3	70.8 ± 0.9
		P2Y4R_Y2_Nter	63.4 ± 2.1 ****	3	96.7 ± 1.4

The constitutive activity is normalized as the ratio of basal relative luminescence units (RFUs) and full activated RFUs, and corresponding values are represented as the mean from three independent biological replicates. Statistical differences in mean constitutive activity are assessed using one-way ANOVA followed by Tukey's multiple comparisons test, comparing each N-terminus swapped mutant to the wild type P2Y2R or P2Y4R. Significance levels are denoted as follows: **** $p < 0.0001$, *** $p < 0.001$, ** $p < 0.01$, * $p < 0.05$. The expression levels of each mutant were measured using a HiBiT assay and were reported as a percentage of the wild-type P2Y2R 's expression level.

Supplementary Table S12. Signaling profiles of N-peptide on P2Y2R N-terminus truncated mutant.

Assay	Ligands	Action	pEC_{50}	E_{max} (%)	n
$G_{s/q}$-based cAMP accumulation	N-peptide	Partial agonist	4.29 ± 0.07	32.2 ± 5.6	6
		Antagonist	4.72 ± 0.04		3
	ATP	Full agonist	6.07 ± 0.04	102.1 ± 1.2	9

The activation of the P2Y2R N-terminus truncated mutant (P2Y2R_Del_Nter) in response to N-peptide and ATP were evaluated. The data are presented as the mean pEC_{50} ± s.e.m. or mean E_{max} ± s.e.m. from three independent replicates, each performed in duplicate or triplicate. The antagonistic effect of the N-peptide on P2Y2R_Del_Nter G_q activation was evaluated under treatment with 3.3 μM ATP. The data are presented as the mean pEC_{50} ± s.e.m. or mean E_{max} ± s.e.m. from three independent replicates.

Supplementary Table S13. Summary of formation probability of hydrogen bonds in ATP-bound and apo P2Y2R-G_q complexes.

Interactions	Complex	Formation probability (%)			
		Run1	Run2	Run3	Mean ± s.e.m.
Y306$^{7.53}$ (O)-N$^{H5.24}$ (ND2)	ATP-P2Y2R-G_q	40.4	34.7	25.5	33.5 ± 4.3
	Apo-P2Y2R-G_q	9.6	4.4	1.6	5.2 ± 2.3
K240$^{6.30}$ (NZ)-D$^{H5.13}$ (CG)	ATP-P2Y2R-G_q	90.3	83.8	70.4	81.5 ± 5.9
	Apo-P2Y2R-G_q	55.3	34.6	64.3	51.4 ± 8.8

The formation probability was evaluated using three runs of molecular dynamics (MD) simulations, each lasting 1μs. The data are presented as the mean ± s.e.m. from three independent replicates.

Supplementary Table S14. Sequence alignment of the last 15 residues of the α5 helix in different G-protein subtypes.

B/W	H5.12	H5.13	H5.14	H5.15	H5.16	H5.17	H5.18	H5.19	H5.20	H5.21	H5.22	H5.23	H5.24	H5.25	H5.26
G_s	R	D	I	I	Q	R	M	H	L	R	Q	Y	E	L	L
G_{i1}	T	D	V	I	I	K	N	N	L	K	D	C	G	L	F
G_o	T	D	I	I	I	A	N	N	L	R	G	C	G	L	Y
G_q	K	D	T	I	L	Q	L	N	L	K	E	Y	N	L	V
miniG$_{s/q}$	K	D	I	I	L	Q	M	N	L	R	E	Y	N	L	V
G_{12}	K	D	T	I	L	Q	E	N	L	K	D	I	M	L	Q
G_{13}	K	D	T	I	L	H	D	N	L	K	Q	L	M	L	Q

The sequence of the α5 helix in the engineered miniG$_{s/q}$ and six representative G-protein subtypes, including G_s, G_{i1}, G_o, G_q, G_{12}, and G_{13}, are compared. Conserved residues are highlighted with an orange background. B/W denotes Ballesteros-Weinstein numbering for G-proteins from GPCRdb (gpcrdb.org).

Supplementary Table S15. Sequence alignment of human P2Y receptors (P2YRs) and interactions between receptor and G-proteins.

	TM2	TM3				ICL2				TM5			TM6							TM7	H8	
B/W	2.37	3.49	3.50	3.53	3.54	34.50	34.51	34.54	34.58	5.61	5.64	5.65	6.29	6.30	6.32	6.33	6.36	6.37	6.40	7.53	8.47	8.48
P2Y1R	S	H	R	G	V	P	L	L	K	I	A	L	L	R	K	S	L	V	V	Y	G	D
P2Y2R	N	H	R	G	V	P	L	L	R	M	R	L	A	K	K	S	T	I	V	Y	G	Q
P2Y4R	D	H	R	G	I	P	L	L	R	M	R	L	S	R	R	S	T	I	V	Y	G	D
P2Y12R	S	D	R	K	T	P	F	S	N	L	E	L	P	R	K	V	K	V	I	Y	C	K
2MeSADP-P2Y1R-G_{11}	$E^{H5.22}$	$Y^{H5.23}$	$Y^{H5.23}$	$N^{H5.19}$	$L^{H5.20}$	$I^{H5.15}$<br>$L^{H5.16}$	$F^{H5.08}$<br>$K^{H5.12}$<br>$I^{H5.15}$	$I^{H5.15}$				$L^{H5.16}$<br>$L^{H5.20}$		$D^{H5.13}$	$V^{H5.26}$		$L^{H5.25}$	$L^{H5.25}$		$N^{H5.24}$	$N^{H5.24}$	$N^{H5.24}$
ATP-P2Y2R-miniG_q	$E^{H5.22}$	$Y^{H5.23}$	$Y^{H5.23}$	$N^{H5.19}$	$L^{H5.20}$	$I^{H5.15}$<br>$L^{H5.16}$	$F^{H5.08}$<br>$K^{H5.12}$<br>$I^{H5.15}$	$I^{H5.15}$	$Y^{H5.23}$	$L^{H5.25}$		$L^{H5.16}$<br>$L^{H5.20}$	$V^{H5.26}$	$D^{H5.13}$	$L^{H5.25}$<br>$V^{H5.26}$	$L^{H5.25}$	$L^{H5.25}$	$L^{H5.25}$		$N^{H5.24}$		
Apo-P2Y2R-miniG_q	$E^{H5.22}$	$Y^{H5.23}$	$Y^{H5.23}$	$N^{H5.19}$	$L^{H5.20}$	$I^{H5.15}$<br>$L^{H5.16}$	$F^{H5.08}$<br>$K^{H5.12}$<br>$I^{H5.15}$	$I^{H5.15}$	$Y^{H5.23}$	$L^{H5.25}$		$L^{H5.20}$	$V^{H5.26}$			$L^{H5.25}$	$L^{H5.25}$	$L^{H5.25}$				
ATP-P2Y2R-miniG_o	$G^{H5.22}$	$C^{H5.23}$	$C^{H5.23}$	$N^{H5.19}$	$L^{H5.20}$	$I^{H5.15}$<br>$I^{H5.16}$	$F^{H5.08}$<br>$T^{H5.12}$<br>$I^{H5.15}$	$I^{H5.15}$<br>$N^{H5.19}$		$L^{H5.25}$	$D^{H5.13}$	$I^{H5.16}$<br>$L^{H5.20}$	$Y^{H5.26}$	$D^{H5.13}$	$Y^{H5.26}$	$L^{H5.25}$	$L^{H5.25}$					
UTP-P2Y4R-miniG_q		$Y^{H5.23}$	$Y^{H5.23}$	$N^{H5.19}$	$L^{H5.20}$	$I^{H5.15}$<br>$L^{H5.16}$	$F^{H5.08}$<br>$K^{H5.12}$<br>$I^{H5.15}$	$I^{H5.15}$		$L^{H5.25}$		$L^{H5.16}$<br>$L^{H5.20}$	$V^{H5.26}$	$D^{H5.13}$	$V^{H5.26}$	$L^{H5.25}$	$L^{H5.25}$	$L^{H5.25}$		$N^{H5.24}$		$N^{H5.24}$
2MeSADP-P2Y12R-G_{i2}			$C^{H5.23}$	$C^{H5.23}$	$L^{H5.20}$	$N^{H5.19}$<br>$I^{H5.15}$<br>$I^{H5.16}$	$F^{H5.08}$<br>$T^{H5.12}$<br>$I^{H5.15}$			$L^{H5.25}$		$I^{H5.16}$<br>$L^{H5.20}$				$F^{H5.26}$	$F^{H5.26}$		$L^{H5.25}$			

The table above shows the sequence alignment of residues from four human P2Y receptors involved in $G_{q/11}$ or $G_{i/o}$ coupling. The table below displays the interaction residues in the α5 helix of G-protein with the corresponding residues in the receptors in the six P2YR-G protein complex, including 2MeSADP-P2Y1R-G_{11} (PDB ID: 7XXH), ATP-P2Y2R-miniG_q, Apo-P2Y2R-miniG_q, ATP-P2Y2R-miniG_o, UTP-P2Y4R-miniG_q, and 2MeSADP-P2Y12R-G_{i2} (PDB ID: 7XXI). Conserved residues with P2Y2R at the same Ballesteros-Weinstein numbering (B/W) position are shown in red. Polar contacts, including hydrogen bonds and salt bridges, are highlighted in blue. Hydrophobic interactions are depicted in black.

Supplementary Table S16. Cryo-EM data collection, refinement and validation statistics.

	ATP-bound P2Y2R-miniG$_q$-Nb35 complex (EMD-61958) (PDB 9K0X)	ATP-bound P2Y2R-miniG$_o$-scFv16 complex (EMD-61986) (PDB 9K20)	apo-bound P2Y2R-miniG$_q$-Nb35 complex (EMD-61990) (PDB 9K25)	UTP-bound P2Y2R-miniG$_q$-Nb35 complex (EMD-61947) (PDB 9K0K)
**Data collection and processing**				
Microscope	Titan Krios G3	Titan Krios G3	Titan Krios G3	Titan Krios G3
Detector	Gatan K3	Gatan K3	Gatan K3	Gatan K3
Magnification	64,000	64,000	64,000	64,000
Voltage (kV)	300	300	300	300
Electron exposure (e–/Å^2)	50	50	50	50
Defocus range (μm)	-1.1~-1.6	-1.1~-1.6	-1.1~-1.6	-1.1~-1.6
Pixel size (Å)	1.08	1.08	1.08	1.08
Symmetry imposed	C1	C1	C1	C1
Initial particle images (no.)	3,378 k	2,137 k	1,697 k	1,850 k
Final particle images (no.)	853 k	273 k	211 k	356 k
Map resolution (Å)	2.83	2.65	3.31	3.14
FSC threshold	0.143	0.143	0.143	0.143
Map resolution range (Å)	50~2.3	50~2.2	50~2.9	50~2.8
**Refinement and validation**				
Initial model used (PDB code)	P2Y2R: P2Y2R homology model (AlphaFold2) miniG$_q$, Gβγ, Nb35: 7RMI	P2Y2R: P2Y2R homology model (AlphaFold2) miniG$_q$, Gβγ, Nb35: 8E9X	This study (PDB: 9K0X)	P2Y4R: P2Y4R homology model (AlphaFold2) miniG$_q$, Gβγ, Nb35: 7RMI
Map sharpening B factor (Å^2)	-143.5	-79.4	-140.5	-133.8
Model composition				
Non-hydrogen atoms	7300	8292	7117	7441
Protein residues	979	1111	976	996
Ligands	ATP	ATP	Ligand-free	UTP
B factors (Å^2)				
Protein	39.75	63.85	82.87	73.98
Ligand	78.42	63.21		69.57
R.m.s. deviations				
Bond lengths (Å)	0.004	0.003	0.004	0.003
Bond angles (°)	0.676	0.656	0.636	0.622
Validation				
MolProbity score	1.52	1.49	1.71	1.77
Clashscore	6.70	6.93	6.98	8.82
Poor rotamers (%)	0.00	0.00	0.00	0.00
Ramachandran plot				
Favored (%)	97.19	97.44	95.39	95.71
Allowed (%)	2.81	2.56	4.61	4.29
Disallowed (%)	0.00	0.00	0.00	0.00
